# Visual adaptation of a biting fly that permanently foregoes flight

**DOI:** 10.1242/jeb.251571

**Published:** 2026-06-02

**Authors:** Roger D. Santer, David C. Wilcockson, Martin T. Swain, Annalisa Andreani, Anita Nencioni, Patrizia Sacchetti

**Affiliations:** ^1^ Department of Life Sciences, Aberystwyth University, Aberystwyth SY23 3DA, UK; ^2^ Department of Agriculture, Food, Environment and Forestry (DAGRI), University of Florence, 50144 Florence, Italy

**Keywords:** Colour vision, Deer ked, Host-seeking, *Lipoptena*, Opsin, Photoreceptor

## Abstract

Sensory systems are essential for behaviour, but energetically expensive. Selection favours accurate coding of pertinent stimuli and penalises excess functionality, but selective pressures can change over an organism's life. The dipteran superfamily Hippoboscoidea includes obligate blood-feeding flies with greatly varying ecologies, from fast-flying, free-living, visually oriented tsetse, to flightless, ectoparasitic, non-visual bat flies. Deer keds (*Lipoptena andaluciensis*, Diptera: Hippoboscidae) combine these lifestyles by flying to seek hosts based at least partly on vision, and then breaking off their wings to live as permanent ectoparasites. We use transcriptomics to understand evolutionary and developmental adaptations of the deer ked visual system to these dramatically varying selective pressures. We identified transcripts for five opsins, corresponding to the visual opsins of free-living tsetse. These included Rh1, Rh3, Rh5 and Rh6 of the compound eyes, comprising UV-, blue- and green-absorbing types serving colour and motion vision, and Rh2 of the ocelli, serving flight control. These opsins were still expressed in wingless, ectoparasitic adults but at significantly reduced levels, consistent with reduced investment in vision but not loss of any aspect of visual function. We speculate that limited plasticity in opsin expression may constrain visual adaptation to ectoparasitism, potentially imposing a long-term cost to foregoing flight.

## Teaser

**Summary:** Winged host-seeking deer ked and wingless ectoparasitic deer ked express the same five opsins as well-studied calyptrate flies, but expression is greatly reduced in wingless keds.

## INTRODUCTION

Sensory systems encode pertinent natural stimuli to drive behavioural responses that enhance fitness, but demand tremendous energetic expenditure, even at rest (Niven and Laughlin, 2008). Therefore, sensory systems are under opposing selective pressures that reward the accurate coding of behaviourally relevant stimuli, whilst penalising unnecessary functionality (Niven and Laughlin, 2008). There are striking examples of evolutionary adaptation of sensory systems in response to these pressures, such as eye loss in Mexican cave fish (Protas et al., 2007). Because costs and benefits are accounted over the life of an organism, and behavioural demands can shift over that time, developmental responses to these pressures are also predicted (e.g. synchronised seasonal changes in opsin expression and behaviour in medaka fish; Shimmura et al., 2017).

Obligate blood feeding flies of the superfamily Hippoboscoidea exhibit great variability in behaviour and ecology, and concomitant visual adaptation. At the extremes, tsetse (Glossinidae) are strongly flying, day-active flies that visit vertebrate hosts only to feed, whilst bat flies (Nycteribiidae and Streblidae) include species with reduced or absent wings, that live under dim light or complete darkness, and rarely leave their hosts (Krinsky, 2019; Petersen et al., 2007; Reeves and Lloyd, 2019). Tsetse possess large compound eyes (Hardie et al., 1989), use colour vision to identify hosts, and are strongly attracted to dark and/or blue objects as a by-product of that mechanism (Gibson and Torr, 1999; Santer et al., 2023). In bat flies, compound eyes and ocelli may be completely absent, or else very greatly reduced (Porter et al., 2021).

Keds and flat flies (Hippoboscidae) comprise the fourth family of Hippoboscoidea and include winged, reduced-winged and wingless species, with variation in the relative size of compound eyes and presence of ocelli (Reeves and Lloyd, 2019; Yatsuk et al., 2024; Zhang et al., 2023). This variation is evident within the evolutionary clade of species specialised for large mammalian hosts, including the Hippoboscinae and Lipopteninae, which contains fully winged, free-flying, large-eyed *Hippobosca* spp. and wingless, ectoparasitic, small-eyed and ocelli-less *Melophagus* spp. (Petersen et al., 2007; Yatsuk et al., 2024). Enigmatic among these are deer ked (*Lipoptena* and *Neolipoptena* spp.) whose adults initially fly to seek hosts, but upon finding a suitable host, break off their wings to live as permanent ectoparasites (Yatsuk et al., 2024; Dibo et al., 2023). Flying adults are attracted to dark and blue objects (Andreani et al., 2021; Kortet et al., 2010), suggesting visually guided host-seeking behaviour reminiscent of tsetse, although their flight is less strong. However, after accepting a host and losing wings, adults live within the fur in a manner reminiscent of bat flies and permanently wingless *Melophagus* spp. keds. This abrupt transition in behavioural demands is not associated with a moult, meaning that changes to external anatomy cannot occur. However, there are substantial physiological adaptations, including histolysis of flight muscles, thickening and displacement of leg muscles, growth of apodemes, and substantial thickening of the cuticle (Schlein, 1967). How the visual system is adapted to serve this peculiar life history is unknown.

The compound eyes of most brachyceran flies that have been studied to date share similarities in anatomy and physiology (e.g. Hardie, 1986; Sharkey et al., 2020). Each ommatidium contains eight photoreceptive retinula cells that comprise five spectral types across most of the compound eye (excluding specialised regions for tracking mates or sensing polarised light) (Hardie, 1986). Outer photoreceptors R1–R6 are broadband-sensitive and express blue–green-absorbing opsin Rh1 (Hardie, 1986; Sharkey et al., 2020). These photoreceptors are believed to serve motion vision (Hardie, 1986). Inner photoreceptors R7 and R8 serve colour vision and occur in y- and p-types found in separate ommatidia (Hardie, 1986). R7p expresses the UV-absorbing opsin Rh3, and R8p expresses the blue-absorbing opsin Rh5 (Hardie, 1986; Sharkey et al., 2020). In *Drosophila*, R7y expresses a second UV-absorbing opsin, Rh4 (Sharkey et al., 2020), but this has been lost in calyptrate flies and UV sensitivity arises instead from a UV-absorbing sensitising pigment that transfers energy to a blue-absorbing opsin, for which blue light absorption is largely blocked by a carotenoid screening pigment (Hardie, 1986; Hardie and Kirschfeld, 1983). R8y expresses the green-absorbing opsin Rh6 (Hardie, 1986; Sharkey et al., 2020). In addition to compound eyes, flies typically possess three ocelli, whose photoreceptors express opsin Rh2 (Pollock and Benzer, 1988). Ocelli are involved in stabilisation reflexes which are particularly important for flight control (Krapp, 2009).

The above pattern appears to be conserved across the majority of calyptrate fly eyes studied to date, with species from the genera *Musca*, *Stomoxys* (both Muscidae) and *Glossina* (Glossinidae) all possessing the same opsin homologues (Attardo et al., 2019; International Glossina Genome Initiative, 2014; Olafson et al., 2021). However, this organisation has been lost in the greatly reduced compound eyes of the bat fly *Trichobius frequens* (Streblidae), which have few facets, variable numbers of retinula cells per ommatidium, and from which only the Rh1 opsin has been identified (Porter et al., 2021). Although little is known about the vision of hippoboscids generally, or deer keds specifically, the closely related but permanently flightless and ectoparasitic sheep ked, *Melophagus ovinus*, lacks the *Rh2* opsin gene, corresponding to its lack of ocelli (Zhang et al., 2023). It has relatively small compound eyes and possesses opsin genes *Rh1*, *Rh3* and *Rh6*, but lacks *Rh5* (Zhang et al., 2023), which might be linked to an absence of ranged host-seeking for which blue–green opponency is believed to be important (Santer et al., 2023).

Here, we investigate the deer ked *Lipoptena andaluciensis* (González et al., 2024). We describe the compound eyes of these flies using scanning electron microscopy. We then characterise opsins and their transcription levels in alate (winged) host-seeking adults caught in flight and dealate (wingless) ectoparasitic adults collected from deer. As opsins mediate photoreceptor responses to light, our focus is on the sensory machinery that most directly permits and shapes visual sensitivity. We hypothesise that the host-seeking behaviour of flying deer keds requires visual machinery aligned to that of tsetse, but that the transition to ectoparasitism should be accompanied by a reduction or cessation of opsin expression owing to the high cost and presumed limited utility of vision in this context. Knowledge of the visual system of deer ked can shed further light on the mechanisms of host-seeking that apply across biting fly species (Gibson and Torr, 1999) and provide a rationale for the development of control or monitoring devices (Santer and Allen, 2024, 2025).

## MATERIALS AND METHODS

### Deer ked sample collection

Deer keds *Lipoptena andaluciensis* (González et al., 2024), of mixed sexes were sampled in the Tuscan Apennines, Italy. Alate, host-seeking adult keds were collected in August 2022, coincident with peak catches of *Lipoptena* spp. in previous work (Andreani et al., 2021). Keds were collected in Schignano (500 m a.s.l.), Prato, Tuscany, along woodland edges and clearings, predominantly by capture into individual vials after alighting on visual lures or the experimenters' clothing. This proved more effective than sweep netting. Dealate ectoparasitic adult keds were collected in March 2023, during the morning, from the carcasses of three red deer, *Cervus elaphus* (two adult females and one juvenile female), that had been killed at dawn of the same day by authorised hunters in wooded areas northwest of Prato, Tuscany (Pistoia province). Synchronised collection of host-seeking and ectoparasitic keds was not possible because collection of ectoparasitic keds could only be done opportunistically and relied on the experimenters being notified of recently killed deer that could be accessed promptly. After collection, host-seeking keds were preserved at intervals throughout the day under field conditions, whereas ectoparasitic keds were taken to the laboratory *en masse* and preserved in the afternoon. In each case, ked heads and bodies were separated using watchmaker's forceps and spring scissors pre-cleaned using RNase-away™ (Thermo Fisher Scientific) and separately preserved in RNA-later™ stabilisation solution (Thermo Fisher Scientific). After transport to the UK, samples were stored frozen at −80°C prior to RNA extraction. Work was approved by the Animal Welfare and Ethical Review Board, Aberystwyth University.

### RNA extraction

Five individual ked heads or bodies respectively were pooled to form a sample. High quality total RNA was extracted using Direct-Zol micro-RNA extraction kits (Zymo Research). Prior to extraction, tissues were ground under liquid N_2_ using two 3 mm stainless steel beads in a pre-cooled Qiagen TissueLyzer twice for 2 min at 50 Hz with application of fresh nitrogen between steps to prevent thawing. Disrupted tissues were then homogenised in 200 μl Tri-reagent for 2 min and for a further 3 min after adding 300 μl Tri-reagent (making a final volume of 500 μl). Extraction then followed manufacturer's guidelines before elution of total RNA in 15 μl DEPC-treated water. Samples were subjected to DNase digestion using 1 μl Turbo DNA-free kits (Thermo Fisher Scientific) for 30 min at 37°C and the reaction terminated using 5 μl DNase Inactivation Reagent. RNA yield was estimated spectrophotometrically using a Nanodrop ND2000 and confirmed and quality checked in an Agilent 2100 Bioanalyzer. We were able to prepare *N*=4 samples for host-seeking ked heads and bodies, *N*=3 for ectoparasitic bodies and *N*=5 for ectoparasitic heads, that had sufficient quality for sequencing. Approximately 1 μg total RNA per sample was submitted for RNA sequencing.

### Transcriptome analysis

Transcriptome sequencing, *de novo* assembly and downstream analyses were performed by BGI Genomics. Trinity (version 2.13.2) was used for assembly (Grabherr et al., 2011); the transcripts were then clustered with CD-HIT (Fu et al., 2012) to identify unigenes (non-redundant sequences) and the quality of the resulting unigene assembly was evaluated with BUSCO (Seppey et al., 2019). Transcript annotation was performed by predicting coding sequences, then peptides, which were searched using Diamond (version 2.0.15.153; Buchfink et al., 2015) against Pfam (Bateman et al., 2004), SwissProt (Boeckmann et al., 2003) and UniProt (UniProt Consortium, 2019), from which the GO terms were obtained. To calculate differentially expressed genes, Bowtie2 (version 2.4.5) (Langmead and Salzberg, 2012) was used to align reads to the unigene assembly, with RSEM (Li and Dewey, 2011) calculating expression levels and DESeq2 (Love et al., 2014) identifying differential expression.

To provide an overview of the transcriptome data, using Gene Ontology (GO) terms we conducted a global analysis of the differentially expressed genes to identify enriched and purified molecular processes (Xiao et al., 2014). This is described and presented in Supplementary Materials and Methods, and Figs S1,S2.

To further investigate opsins specifically, all transcripts with annotations that contained the search term ‘opsin’ were extracted from the unigene assembly (*N*=102 in total) and opsins were identified by their alignment to UniProt *Drosophila* opsin sequences (*N*=11; Figs S5, S6). Predicted protein sequences were investigated using DeepTMHMM (Hallgren et al., 2022 preprint) to ascertain completeness based upon possession of seven transmembrane domains (Table S1).

Predicted opsin protein sequences deemed complete were aligned using MUSCLE (Edgar, 2004) to opsin protein sequences for *Drosophila melanogaster*, *Glossina* spp., *Musca domestica* and *Stomoxys calcitrans* obtained from Vectorbase (Giraldo-Calderón et al., 2022). Phylogenetic analysis was conducted using MEGA 12 (Kumar et al., 2024). Relative transcription levels were quantified using read counts, TPM (transcripts per million) and FPKM (fragments per kilobase of transcript per million mapped reads). Analyses based on TPM are provided in text, with others presented in Table S2.

### Scanning electron microscopy and species confirmation

Alate host-seeking keds were collected from the same locations in 2024 and preserved in 70% ethanol. Keds (males and females, ca. 20 specimens in total) were sonicated for 5 min in 70% ethanol and dehydrated using a graded ethanol series (80%, 90%, 95% and 99%) for 10 min per concentration. After dehydration, entire keds and separated heads were mounted in different positions onto stubs and observed, without coating, by scanning electron microscope (Hitachi SU8700) at the Center for Electron Microscopy and Microanalysis Services (MEMA), University of Florence.

*Lipoptena andaluciensis* is newly described (González et al., 2024) and the identity of deer ked sampled in Italy has since been discussed. *Lipoptena andaluciensis* has been confirmed from Emilia-Romagna Region on the basis of morphological features and CO1 DNA barcoding (Usai et al., 2025). To confirm the identity of keds in our sample, we searched the unigene assembly for deer ked CO1 sequences obtained from NCBI GenBank, finding a 100% coverage, 100% identity match for *L. andaluciensis* sequence PQ176810 (González et al., 2024). Furthermore, SEM images revealed thoracic chaetotaxy diagnostic of *L. andaluciensis* (González et al., 2024; Usai et al., 2025).

## RESULTS

**Fig. 1.**
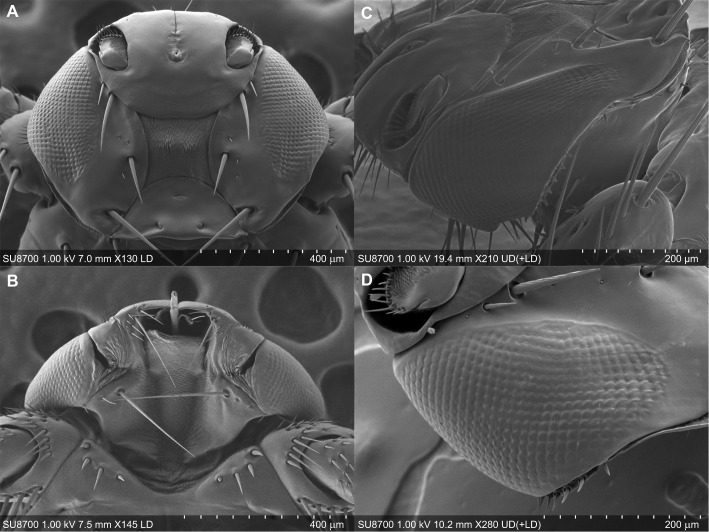
**Scanning electron micrographs showing the head of the deer ked *Lipoptena andaluciensis*.** (A–C) Dorsal (A), ventral (B) and lateral (C) views of the head. (D) Dorsal view of the left compound eye. The anterior direction is towards the top of the figure in A and B, and left of the figure in C and D. Each image is of a different individual.

The heads of alate host-seeking deer keds are wider than they are long and dorsoventrally flattened (Fig. 1A–C). Three ocelli are present in triangular arrangement on the posterior part of the dorsal surface (Fig. 1A). Each compound eye is 220–260 μm wide at the anterolateral margin of the head, and wraps around it to cover its outer dorsal and ventral surfaces up to ca. 25% of head width at the widest point (Fig. 1A,B). The dorsal part of the compound eye is relatively flattened (Fig. 1C). Compound eye facets are hexagonal anteromedially with diameters of ca. 15 μm but become square laterally and dorsally (anteromedial diameters: 14.9±0.9 μm; dorsal diameters: 13.8±0.6 μm; means±s.d.; *n*=10 well-resolved facets in Fig. 1D). We estimate that each compound eye has approximately 500–550 facets in total, based on three dorsal and three ventral SEM images, each from a different individual ked. Keds become dealate ectoparasites without a moult that could modify these outer cuticular structures, so we investigated photoreceptor-level adaptations using transcriptomics.

The completeness of the *L. andaluciensis* transcriptome was assessed using benchmarking universal single-copy orthologs (BUSCO) analysis to assess the presence of expected genes. We achieved a completeness score of 97.7%. Of 255 BUSCO groups searched, 249 were complete, with four fragmented and two missing. Genes associated with GO terms muscle contraction, muscle structure and development, compound eye photoreceptor development, digestion, oogenesis and male courtship behaviour (among others) were significantly enriched among genes differentially expressed in host-seeking and ectoparasitic deer ked, aligning with expectations based on life history (Figs S1,S2).

**Fig. 2.**
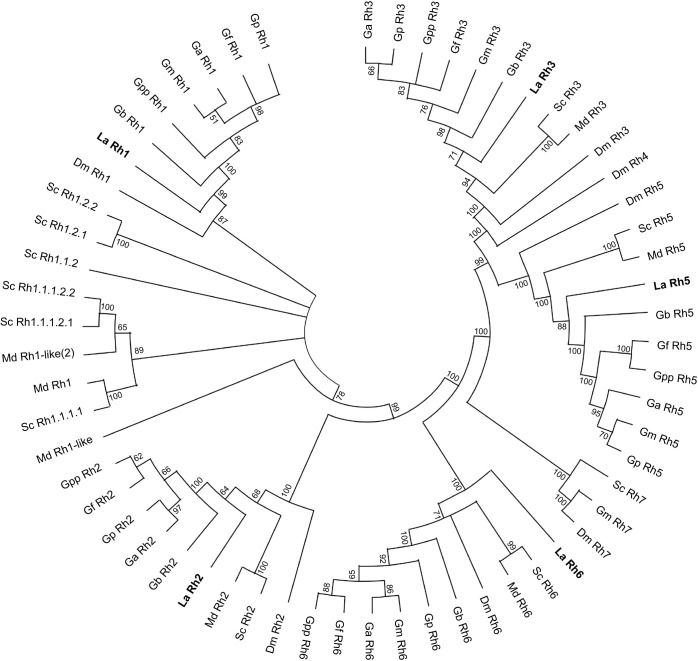
**Evolutionary relationship of previously identified fly opsin proteins and those predicted from deer ked opsin transcripts.** Bootstrap consensus tree inferred from 1000 replicates constructed using the neighbour-joining method. Bootstrap values shown at nodes. La, *Lipoptena andaluciensis*; Dm, *Drosophila melanogaster*; Ga, *Glossina austeni*; Gb, *Glossina brevipalpis*; Gf, *Glossina fuscipes*; Gm, *Glossina morsitans*; Gp, *Glossina pallidipes*; Gpp, *Glossina palpalis*; Md, *Musca domestica*; Sc, *Stomoxys calcitrans*. Deer ked opsin sequences are highlighted in bold and cross-referenced to the Trinity assembly in Table S1.

We identified seven transcripts that matched *Drosophila* opsin sequences and had predicted protein sequences with the expected seven transmembrane domains, and a further three that appeared to be fragments of these (Table S1). By alignment of the seven complete predicted protein sequences against those for *D. melanogaster*, *M. domestica*, *S. calcitrans* and six *Glossina* species, we identified homologues of Rh1, Rh2, Rh3, Rh5 and Rh6 (Fig. 2). Two transcripts had predicted protein sequences that were identical and aligned to Rh1, and two transcripts had predicted protein sequences that differed only by inclusion or omission of 25 initial amino acid residues and were aligned to Rh2 (Table S1). We did not identify a homologue of *Drosophila* Rh4 or Rh7.

**Fig. 3.**
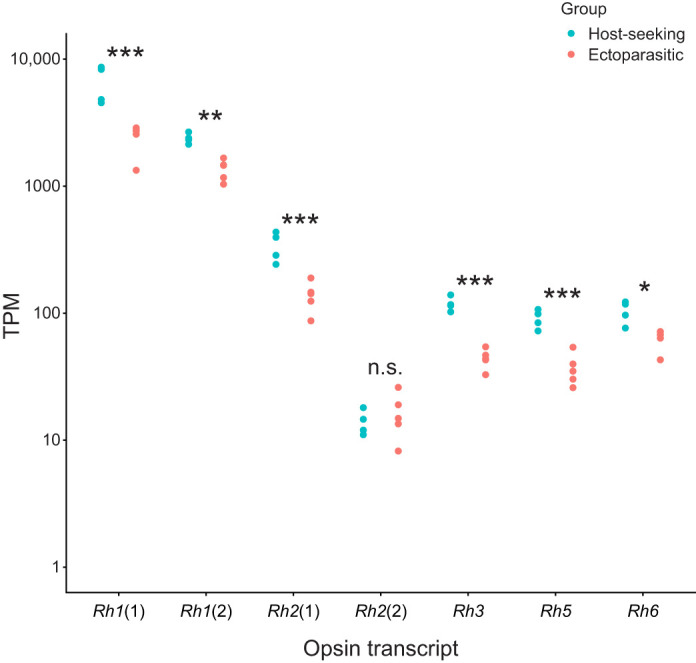
**Transcripts per million (TPM) values for each of the complete opsin transcripts in deer ked heads.** Note that these are plotted on a log scale. *N*=4 replicates for alate host-seeking keds; *N*=5 replicates for dealate, ectoparasitic keds, each replicate pooling five flies. Asterisks indicate significant differences between host-seeking and ectoparasitic keds according to Wald tests implemented using DESeq2 (Love et al., 2014; see Table 1); n.s., *P*>0.05, *P<0.05, **P<0.01, ***P<0.001.

Each of the seven transcripts associated with a complete protein sequence was retrieved from both host-seeking and ectoparasitic deer ked heads (Fig. 3), but not from host-seeking or ectoparasitic deer ked bodies (mean TPM generally <1 and always <4; Table S2). Abundance of both *Rh1* transcripts was more than ten times greater compared with *Rh3*, *Rh5* and *Rh6* transcripts (Fig. 3). The *Rh2* transcript that resulted in the shorter predicted protein sequence was least common (Fig. 3). Expression of each opsin in host-seeking deer keds was approximately double that in ectoparasitic keds, although this difference was less marked for *Rh6* (Fig. 3, Table 1). Expression was significantly different in all cases except the rarer duplicate *Rh2* transcript that was associated with the shorter predicted protein sequence (Table 1). However, this difference was no longer significant for *Rh6* when a false discovery rate correction was applied at the level of the whole transcriptome (Table 1).

**Table 1.** Analysis of differential opsin expression using DESeq2 (Love et al., 2014), comprising log_2_ fold change (FC) for host-seeking compared with ectoparasitic keds, Wald test *P*-value and false discovery rate at the level of the complete transcriptome (*q*-value)

Opsin transcript	log_2_ FC	*P*-value	*q*-value
*Rh1(1)*	1.164	**<0.001**	**0.001**
*Rh1(2)*	0.555	**0.002**	**0.013**
*Rh2(1)*	1.043	**<0.001**	**<0.001**
*Rh2(2)*	−0.456	0.168	0.349
*Rh3*	1.177	**<0.001**	**<0.001**
*Rh5*	1.040	**<0.001**	**<0.001**
*Rh6*	0.442	**0.043**	0.125

## DISCUSSION

In this work we characterised the opsin sequences of alate, host-seeking and dealate, ectoparasitic deer keds, *Lipoptena andaluciensis*, aiming to identify evolutionary and developmental adaptations to their peculiar life history. We identified the five opsins typical of calyptrate fly compound eyes and ocelli, indicating a visual system similar to that of tsetse. However, we found that expression of all these opsins was significantly reduced to about 50% of prior levels after the transition to ectoparasitism, suggesting reduced investment in vision but not a loss of any aspect of visual function, at least at the transcription level.

The compound eye of the deer ked forms a flattened surface that curves around the anterolateral margins of the head. Facet shapes transition from hexagonal anteromedially to square laterally and dorsally. This resembles part of the transition in facet shape seen in *Musca* spp. and *Calliphora* spp., which maintains a hexagonal array of visual axes despite the changing curvature of the eye (Stavenga, 1975; Hardie, 1985). The different pattern in deer keds probably reflects the different shape of the eye, itself necessitated by the dorsoventrally flattened body plan adaptive for ectoparasitism. The implications of this for vision will be an interesting topic for future investigation. In addition, the deer ked compound eye has <550 facets in total, fewer than seen in *Drosophila* (ca. 700), *Musca* spp. (3000) or *Calliphora* spp. (5000), and facet diameters in deer ked appear to be smaller than in those species (Hardie, 1985).

The opsin proteins of *L. andaluciensis* aligned to the known opsin types of tsetse (*Glossina* spp.), stable flies (*S. calcitrans*) and house flies (*M. domestica*) (Attardo et al., 2019; International Glossina Genome Initiative, 2014; Olafson et al., 2021). A single Rh1 opsin was identified and the adundance of the two associated transcripts was more than ten times greater than transcripts for opsins Rh3, Rh5 and Rh6, reflecting the expression of this opsin in six out of eight retinula cells in most ommatidia of a typical calyptrate fly compound eye (Hardie, 1986). The significance of the two transcripts identified is unclear, given that the genome of *L. andaluciensis* has not yet been sequenced. Possibilities include alternative splicing, between-individual genetic polymorphism, or even sequencing or assembly error (Fig. S3). In addition, multiple *Rh1* homologues are present in the genomes of *S. calcitrans* and *M. domestica*, albeit that in *S. calcitrans* only one of these was expressed at high levels (Olafson et al., 2021). Regardless, both transcripts were abundant and appear to encode identical proteins. We did not identify a homologue of the *Drosophila* Rh4 opsin, reinforcing the pattern of loss seen in all calyptrate flies studied to date (Attardo et al., 2019; International Glossina Genome Initiative, 2014; Olafson et al., 2021). However, we did identify transcripts encoding opsins Rh3, Rh5 and Rh6, suggesting that *L. andaluciensis* has an intact colour visual system similar to that of tsetse and that none of these opsins has been lost as in the closely related but permanently ectoparasitic and flightless sheep ked, *M. ovinus* (Petersen et al., 2007; Yatsuk et al., 2024; Zhang et al., 2023). In addition, we identified relatively high expression of the ocellar opsin Rh2, probably reflecting the need for ocelli in flight control (Krapp, 2009). We identified two *Rh2* transcripts, but one was characterised by an 87 bp insertion (Fig. S4), was far less common, did not differ in expression level between life stages and resulted in a shorter predicted protein sequence. Potential explanations are as described for *Rh1* above, although in this case it is conceivable that the resulting protein would not be functional since mutations of N- and C-terminal tails of vertebrate rhodopsin are associated with disease (Athanasiou et al., 2018). We did not identify a homologue of *Drosophila* Rh7, which is associated with non-visual photoreception. Thus, it appears that *L. andaluciensis* possesses a similar visual system to free-living and fast-flying tsetse flies, which probably supports the visually guided host seeking of newly eclosed *Lipoptena* spp. adults (Andreani et al., 2021; Kortet et al., 2010).

A blowfly retina is estimated to account for ca. 10% of the fly's total oxygen consumption (Howard et al., 1987). Given this high metabolic cost, it is unsurprising that some permanently ectoparasitic flies have much smaller or absent compound eyes and ocelli, and have lost opsins, as in the previously mentioned sheep ked *M. ovinus* (Petersen et al., 2007; Yatsuk et al., 2024; Zhang et al., 2023). Since the permanent transition of adult deer keds to ectoparasitism is not associated with a moult, there can be no change to external features of the compound eye or ocelli. However, *L. andaluciensis* exhibits a reduction in the expression of all opsins which was roughly symmetrical across the different opsin types, albeit that reduction in expression of green-absorbing opsin Rh6 was less marked than for the other types. This is consistent with a reduced investment in vision, potentially due both to its limited utility for ectoparasitic adults, and the need to invest in other functions in that stage, including digestion and reproduction (Figs S1,S2). Similar results have been reported in medaka fish, which reduce opsin expression in their less active winter season and increase it during their summer courtship season where attraction to coloured signals occurs (Shimmura et al., 2017). However, a variety of environmental stimuli can trigger changes in opsin expression, including light levels (Shimmura et al., 2018) and circadian rhythm (Li et al., 2020). Ked flight periods restricted flying ked collection to August and technical and logistical issues in accessing recently hunted deer restricted ectoparasitic ked collection to March. Furthermore, logistical constraints necessitated small differences in ked handling, such as time confined to vials before processing and use of artificial light during that task. Therefore, we cannot rule out the possibility that additional environmental factors may have contributed to the patterns observed.

On its own, decreased opsin expression would be expected to reduce the concentration of photopigment in rhabdomeres, reducing the availability of photopigment molecules for photoisomerization and thus sensitivity to light. Opsin expression levels are directly positively related to sensitivity to optomotor stimuli and male signalling colours in guppies (Sakai et al., 2016, 2018). In addition, decreased photopigment concentration would affect spectral sensitivity by reducing self-screening, narrowing a photoreceptor's spectral sensitivity function (see Stavenga et al., 2000). However, internal changes to photoreceptor arrays have been documented in a variety of insects and are thus feasible in deer keds. In locusts, fluctuations in photoreceptor membrane turnover during a day cause cross sectional area of the distal rhabdom to increase at dusk and decrease at dawn, resulting in increased sensitivity but decreased spatial acuity during the dark period (Williams, 1983). If such changes occurred uniformly in ectoparasitic deer keds, they might permit photopigment concentration to be maintained at the cost of reducing the cross-sectional area or length of the rhabdom, then reducing sensitivity through reduced light acceptance angle or a reduced number of individual microvilli available to respond to light. However, a more intriguing possibility is that all or some of these modifications could occur non-uniformly, maintaining performance in sparsely distributed photoreceptors at the cost of compromised spatial acuity, or in specific eye regions at the cost of reduced field of view. Further research will be required to explore these possibilities.

Colour vision depends upon neural comparison of responses in different photoreceptor types. Given the evolutionary loss of opsins Rh2 and Rh5 in the related sheep ked (Zhang et al., 2023), the loss of compound eyes and ocelli in many bat flies (Porter et al., 2021) and the extensive physiological changes that deer ked undergo after host selection (Schlein, 1967), it is perhaps intriguing that *L. andaluciensis* continues to express all of its opsins as a dealate ectoparasite. Since ectoparasitic ked were caught before the flight period, we can rule out the possibility that they had recently settled, and thus we presume that opsin expression reflects normal constitutive expression in ectoparasitic keds. By contrast, different odorant binding proteins and chemoreceptors are down- or upregulated in female *Anopheles gambiae* mosquitoes following a blood meal, resulting in shifts in relative sensitivity to different odorants that can be explained by a shift from host-seeking to oviposition behaviour (Fox et al., 2001; Rinker et al., 2013). In many insects, shifts in the opsins expressed occur between life stages where behavioural demands are very different (e.g. dragonflies; Futahashi et al., 2015). In *Drosophila* pupae, opsin mRNAs are present at low levels much earlier in development than proteins can be detected (Earl and Britt, 2006), so the presence of opsin mRNAs does not always predict translation to opsin proteins (McCulloch et al., 2022). Nevertheless, our data are consistent with generally reduced investment in vision, but not a loss or reconfiguration of any particular aspect of visual function (e.g. colour vision generally, blue–green opponency specifically or ocellar photoreception). One possibility is that a standard calyptrate fly visual system with capacity for colour vision remains useful to ectoparasitic deer keds on their host, or in the case that they are dislodged and need to seek a new host on foot. Alternatively, it may be that there is limited plasticity in opsin expression within the adult stage, as has been observed in sticklebacks subject to changing light conditions (Flamarique et al., 2013). If this were the case, foregoing flight in the manner that deer keds do could incur a considerable long term energetic cost through maintaining visual machinery with limited utility, potentially helping to explain the comparative rarity of this life history among Hippoboscoidea (Yatsuk et al., 2024).

In a wider sense, deer keds provide an ideal model system for understanding the generalised mechanisms of host seeking in diurnal biting flies through comparison of host-seeking and ectoparasitic adults, owing to the unique separation of behaviour between those stages. Greater investment in an intact calyptrate fly visual system in host-seeking adults emphasises the importance of vision in host-seeking (Gibson and Torr, 1999; Santer et al., 2023), and knowledge of that visual system provides a rationale for the development of improved control or monitoring devices (Santer and Allen, 2024, 2025).

## Supplementary Material

10.1242/jexbio.251571_sup1 Supplementary information
