# Supplementary material for: Visual adaptation of a biting fly that permanently foregoes flight
Source: J Exp Biol. 2026 Jun 2;229(10):jeb251571. doi: 10.1242/jeb.251571 (PMC13286350; doi:10.1242/jeb.251571)
Supplement: Supplementary information [file jexbio-229-251571-s1.pdf]

GO Term Analysis

In order to evaluate overall differences in the transcriptomes of host-seeking and ectoparasitic deer keds, *Lipoptena andaluciensis*, we undertook an analysis of GO terms. We combined our separate head and body samples and computed a score based on log2 fold-change and differential expression p-values. This was used to rank the differentially expressed genes, following the approach suggested by Xiao et al. (2014):

$$\text{score} = -1 * \log2(\text{fold change}) * \log10(\text{p value})$$

We defined highly differentially expressed genes as those for which this score was >|10|, combining those that were up- and down-regulated between the two samples. By comparison of this set of differentially expressed genes to the set of all genes in the transcriptome using GOAtools (Klopfenstein et al., 2018), we identified GO classifications that were significantly over-represented (enriched) or significantly under-represented (purified). The GO terms were then visualised using REVIGO (Supek et al., 2011). The results are presented here using low-level GO terms (figures S1-S2).

After their final moult, deer keds fly to seek hosts, but upon finding a suitable host break off their wings to live as ectoparasites (Dibo et al., 2023). Consequently, it is only alate, host-seeking adults that fly and actively seek hosts, and it is only dealate, ectoparasitic adults that digest blood meals and reproduce. The low-level GO terms of genes enriched in our transcripts reflected that dichotomy since they demonstrate that genes associated with muscle contraction, and muscle structure and development; compound eye photoreceptor development; digestion; oogenesis, and male courtship behaviour, were significantly over-represented among genes that were differentially expressed between host-seeking and ectoparasitic deer keds (figure S1). Meanwhile, genes associated with fundamental metabolic processes common to both life stages were purified (figure S2).

Journal of Experimental Biology • Supplementary information

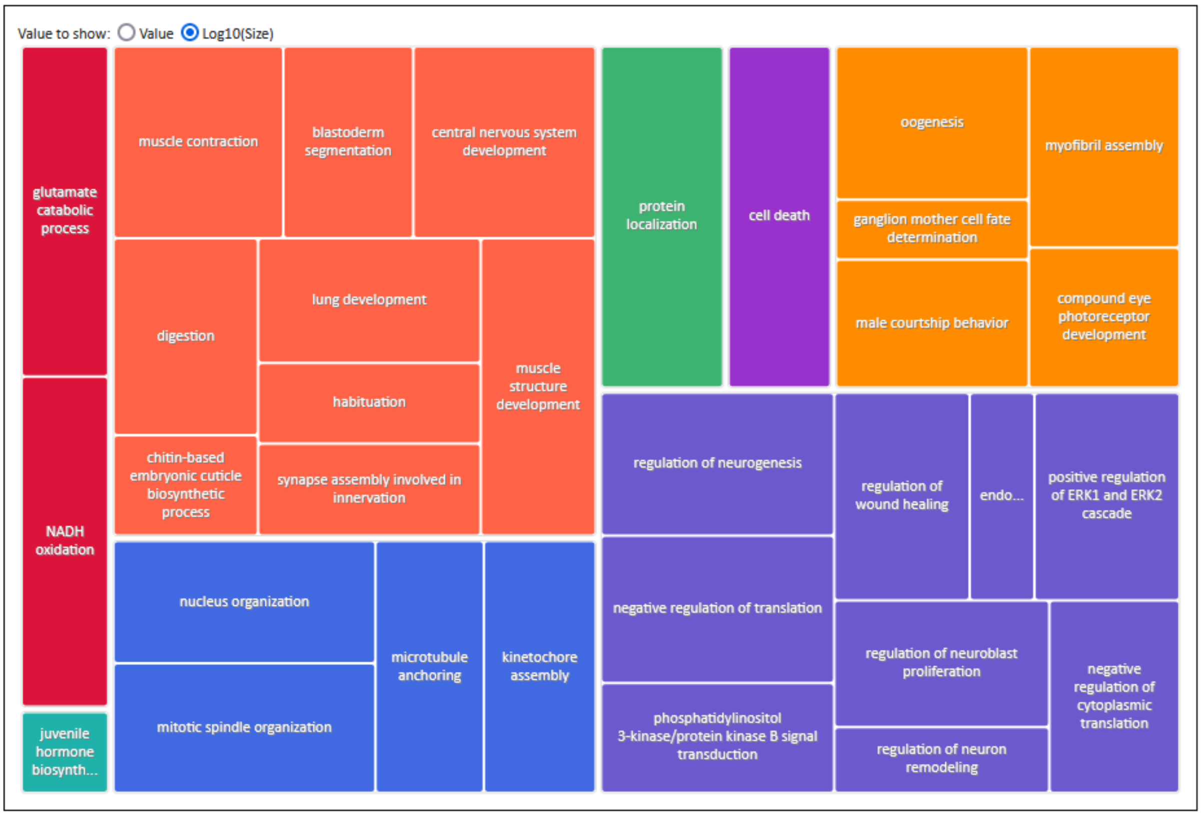

Fig. S1. Low-level GO terms of genes that were significantly enriched among those genes that were differentially expressed between host-seeking and ectoparasitic deer keds, *Lipoptena andaluciensis*.

Journal of Experimental Biology • Supplementary information

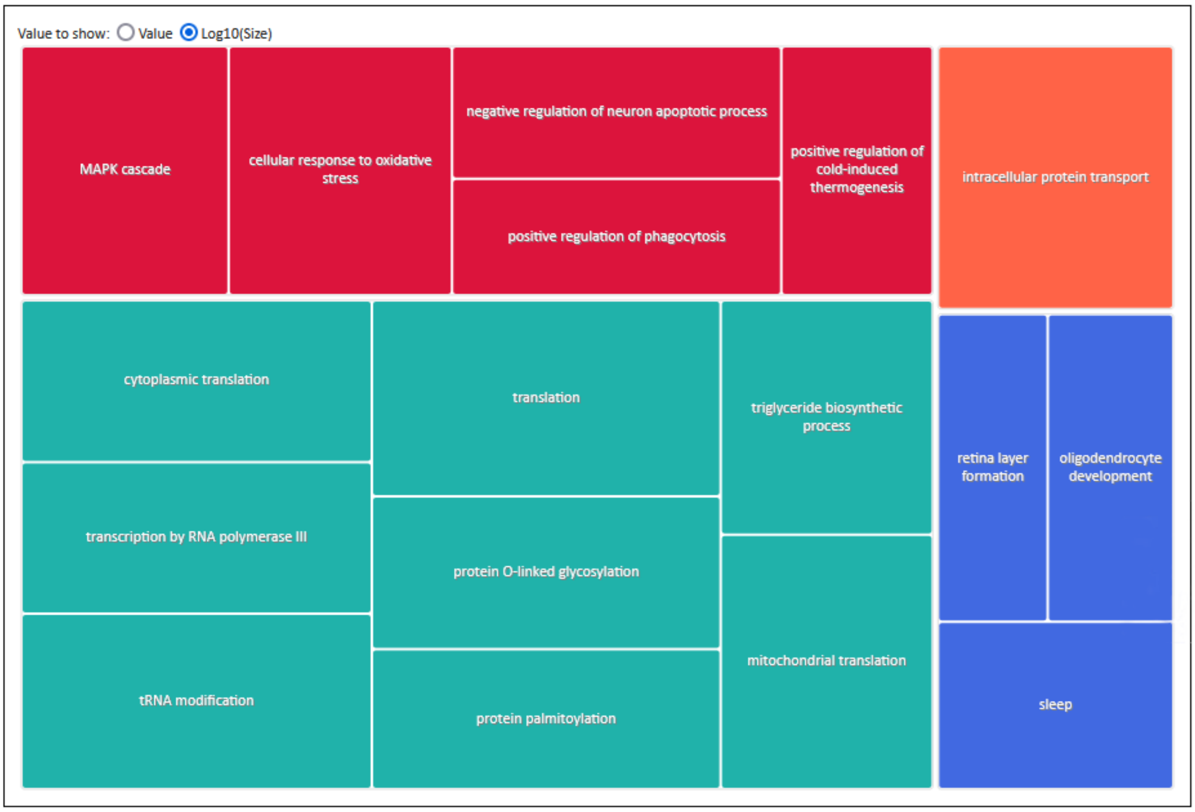

Fig. S2. Low-level GO terms of genes that were significantly purified among those genes that were differentially expressed between host-seeking and ectoparasitic deer keds, *Lipoptena andaluciensis*.

Analysis of Opsin Transcripts

In total, 102 transcripts were extracted from the transcriptome based on their annotations using the search term 'opsin'. Of these, 11 transcripts were matched to *Drosophila* opsin sequences from UniProt (table S1).

Predicted protein sequences for these 11 transcripts were investigated using DeepTMHMM (Hallgren et al., 2022), and seven were determined to be complete based upon inclusion of seven expected transmembrane domains (table S1). Of these seven, two with UniProt hits to Rh1 were identical, and two with Uniprot hits to Rh2 differed only by inclusion or omission of 25 initial amino acid residues (table S1, appendix S2).

Of the four incomplete protein sequences, three were determined to be fragments of the complete Rh3 by blastp alignment, including a short sequence originally matched to *Drosophila* Rh4 on UniProt (table S1). An incomplete sequence matched to Rh1 was not well matched to

Journal of Experimental Biology • Supplementary information

the complete Rh1 sequences, but was only recovered from one sample out of 16 and was thus discarded as anomalous.

Therefore, we identified complete sequences for opsins Rh1, 2, 3, 5, and 6, and consider that the partial sequences are incomplete fragments of these.

To investigate opsin expression, we focused on the seven transcripts associated with complete protein sequences. By pairwise alignment using EMBOSS needle (Rice et al., 2000), we found that the two transcripts resulting in identical protein sequences aligned to Rh1 shared a 1243-base region with 99% identity (3 mismatches) (figure S3). Both transcripts were abundant in our samples (table S2). In *Stomoxys calcitrans* and *Musca domestica*, multiple, clustered Rh1 homologues are present in the genome, but only one is expressed at high levels according to RNA-seq data (Olafson et al., 2021). Thus, multiple Rh1 genes are conceivable, but high expression of both is unexpected. Additional explanations include alternative splicing, and between-individual variation in the Rh1 gene itself. Regardless, because both transcripts code identical proteins, we assume that this variation is not functionally significant, and suggest that genome sequencing may in future shed light on the issue. The two transcripts resulting in nearly identical protein sequences aligned to Rh2 shared 264-base and 1263-base regions with 100% identity, with an additional 87-base region in between in the transcript that resulted in the shorter protein sequence (figure S4). Potential explanations for this are as discussed for Rh1 transcripts. However, in this case the shorter predicted protein sequence omitted 25 initial amino acid residues, and in vertebrates, mutations to N- and C-terminal tails of rhodopsin are associated with disease (Athanasiou et al., 2018). Coupled with the fact that this transcript was notably less abundant in our samples (table S2), it is conceivable that this represents a non-functional form. For each of the complete opsin transcripts, expression was greatest in deer ked heads, and negligible in deer ked bodies (table S2). Expression was also greater in alate, host-seeking than dealate, ectoparasitic ked heads, and these trends were robust to the expression metrics used (table S2).

Journal of Experimental Biology • Supplementary information

Table S1. Putative opsin transcripts recovered from *Lipoptena andaluciensis*. TMDs = transmembrane domains.

Transcript ID	UniProt hit	TMDs	Notes	Opsin
TRINITY_DN7305_c0_g1_i4-s2_head	[OPS1_DROME|diamond] RecName: Full=Opsin Rh1; AltName: Full=Neither inactivation nor afterpotential E protein; AltName: Full=Outer R1-R6 photoreceptor cells opsin;	7	Protein sequence identical to *TRINITY_DN2730_c0_g2_i6-s3_head* [a]	Rh1(1)
TRINITY_DN2730_c0_g2_i6-s3_head	[OPS1_DROME|diamond] RecName: Full=Opsin Rh1; AltName: Full=Neither inactivation nor afterpotential E protein; AltName: Full=Outer R1-R6 photoreceptor cells opsin;	7	Protein sequence identical to *TRINITY_DN7305_c0_g1_i4-s2_head* [a]	Rh1(2)
TRINITY_DN8493_c0_g1_i1-w2_head	[OPS1_CALVI|diamond]RecName: Full=Opsin Rh1; AltName: Full=Outer R1-R6 photoreceptor cells opsin;	6	Not well matched to *TRINITY_DN2730_c0_g2_i6-s3_head* [b], but read count 0 in all but one sample	-
TRINITY_DN1081_c0_g2_i1-s4_head	[OPS2_DROME|diamond] RecName: Full=Opsin Rh2; AltName: Full=Ocellar opsin;	7	Protein sequence identical to *TRINITY_DN1886_c0_g3_i2-w3_head* [c]	Rh2(1)
TRINITY_DN1886_c0_g3_i2-w3_head	[OPS2_DROME|diamond] RecName: Full=Opsin Rh2; AltName: Full=Ocellar opsin;	7	Protein sequence identical to *TRINITY_DN1081_c0_g2_i1-s4_head* [c]	Rh2(2)
TRINITY_DN2863_c0_g3_i3-s1_head	[OPS3_DROPS|diamond] RecName: Full=Opsin Rh3; AltName: Full=Inner R7 photoreceptor cells opsin;	7		Rh3
TRINITY_DN2863_c0_g3_i2-s1_head	[OPS4_DROME|diamond] RecName: Full=Opsin Rh4; AltName: Full=Inner R7 photoreceptor cells opsin;	4	Incomplete fragment of *TRINITY_DN2863_c0_g3_i3-s1_head* [d]	Rh3 (partial)
TRINITY_DN7379_c0_g4_i3-s2_head	[OPS3_DROPS|diamond] RecName: Full=Opsin Rh3; AltName: Full=Inner R7 photoreceptor cells opsin;	4	Incomplete fragment of *TRINITY_DN2863_c0_g3_i3-s1_head* [e]	Rh3 (partial)

Journal of Experimental Biology • Supplementary information

TRINITY_DN1981_c1_g1_i1-w1_head	[OPS4_DROME|diamond] RecName: Full=Opsin Rh4; AltName: Full=Inner R7 photoreceptor cells opsin;	3	Incomplete fragment of *TRINITY_DN2863_c0_g3_i3-s1_head* [f]	Rh3 (partial)
TRINITY_DN1244_c0_g1_i1-w1_head	[OPS5_DROME|diamond] RecName: Full=Opsin Rh5;	7		Rh5
TRINITY_DN435_c0_g5_i1-w4_head	[OPS6_DROME|diamond] RecName: Full=Opsin Rh6; AltName: Full=Rhodopsin Rh6, long-wavelength;	7		Rh6

[a] Query cover: 100%, e value: 0.0, Per. Ident.: 100%.

[b] Query cover: 100%, e value: 7e-164, Per. Ident.: 76.6%, query length: 281, subject length: 384.

[c] Query cover: 93%, e value: 0.0, Per. Ident.: 100%; identical after second initiator in longer sequence.

[d] Query cover: 81%, e value: 1e-138, Per. Ident.: 99.46%, query length: 226, subject length: 384.

[e] Query cover: 84%, e value: 4e-153, Per. Ident.: 99.01%, query length: 243, subject length: 384.

[f] Query cover: 100%, e value: 1e-111, Per. Ident.: 100%, query length: 149, subject length: 384.

Table S2. Quantification of opsin expression in host-seeking and ectoparasitic deer ked heads and bodies, by different metrics. TPM = Transcripts Per Million; FPKM = Fragments Per Kilobase of transcript per Million mapped reads.

Metric	Opsin	Host-seeking (body)	**Host-seeking (head)**	Ectoparasitic (body)	**Ectoparasitic (head)**
TPM	Rh1(1)	1.62±1.91	**6570.86±2201.48**	0.67±0.38	**2449.78±633.96**
	Rh1(2)	0.61±0.75	**2378.52±221.48**	0.21±0.11	**1359.13±253.17**
	Rh2(1)	0.11±0.06	**339.52±90.55**	3.88±3.2	**137.75±37.03**
	Rh2(2)	0.02±0.05	**13.91±3.13**	0.74±0.82	**16.3±6.66**
	Rh3	0±0	**118.28±15.28**	0.12±0.11	**44.04±7.75**
	Rh5	0.12±0.09	**90.53±15.33**	0.15±0.19	**36.95±10.8**
	Rh6	0.07±0.05	**103.25±21.22**	0.02±0.02	**63.42±11.93**
FPKM	Rh1(1)	1.46±1.74	**5835.06±1510.79**	0.87±0.47	**2719.5±714**
	Rh1(2)	0.55±0.68	**2171.11±376.71**	0.28±0.14	**1508.76±291.24**
	Rh2(1)	0.1±0.05	**303.05±59.57**	5.19±4.27	**152.94±41.92**
	Rh2(2)	0.02±0.04	**12.84±4.17**	0.99±1.12	**18.08±7.35**
	Rh3	0±0	**107.65±18.09**	0.15±0.14	**48.84±8.58**
	Rh5	0.1±0.07	**81.35±8.67**	0.19±0.25	**40.98±12.08**
	Rh6	0.06±0.04	**92.46±12.22**	0.03±0.03	**70.4±13.69**
Reads	Rh1(1)	30.59±36.11	**118322.68±30704.57**	18.72±8.92	**54410.45±14141.59**
	Rh1(2)	26.41±32.18	**100050.43±16690.2**	13.61±6.31	**68420.8±12954.13**
	Rh2(1)	2.27±1.25	**6877.68±1343.2**	130.13±106.88	**3422.13±920.51**
	Rh2(2)	0.49±0.97	**310.57±98.93**	27.2±31.42	**430.67±169.88**
	Rh3	0±0	**2490.51±393.66**	3.65±3.43	**1115.32±184.9**
	Rh5	3±2.16	**2234±229.57**	5.33±6.81	**1107.6±316.43**
	Rh6	2.16±1.61	**3216.95±424.32**	1±1	**2410.93±467.13**

```
#========================================
#
# Aligned_sequences: 2
# 1: TRINITY_DN7305_c0_g1_i4-s2_head
# 2: TRINITY_DN2730_c0_g2_i6-s3_head
# Matrix: EDNAFULL
# Gap_penalty: 10.0
# Extend_penalty: 0.5
#
# Length: 2879
# Identity:    1368/2879 (47.5%)
# Similarity:  1368/2879 (47.5%)
# Gaps:        1507/2879 (52.3%)
# Score: 6767.5
#
#
#========================================

TRINITY_DN730      1 ATTTTTTGTTTGAAATTTTTAAATTTCTGCAAACAATAAGAAAGTAATTG     50
                                     ||||||||||||||||||||||||||||||||||
TRINITY_DN273      1 ----------------TTTTAAATTTCTGCAAACAATAAGAAAGTAATTG     34

TRINITY_DN730     51 AGCAGACAAGTGCATAAAAAAAGGTATAAATTTGTTAACGTACATCAATA    100
                     |||||||||||.||| ||||||||||||||||||||||||||||||||||
TRINITY_DN273     35 AGCAGACAAGTACAT-AAAAAAGGTATAAATTTGTTAACGTACATCAATA     83

TRINITY_DN730    101 GGTTCCGCAGGTTTGAATATAACGGCGCCACGAATAGTCCTCATCG----    146
                     ||||||||||||||||||||||||||||||||||||||||||||||
TRINITY_DN273     84 GGTTCCGCAGGTTTGAATATAACGGCGCCACGAATAGTCCTCATCGGTAA    133

TRINITY_DN730    147 --------------------------------------------------    146

TRINITY_DN273    134 TTTCTCAAATAAAGTGCCAAAAATCAAATAAATGAAACTAGCAAATTGTG    183

TRINITY_DN730    147 --------------------TAAATAATTTAGGAATTTTTCGTTAAACGT    176
                                         ||||||||||||||||||||.|||||||||
TRINITY_DN273    184 ACGAAATTTAAACAATACAATAAATAATTTAGGAATTTTTGGTTAAACGT    233

TRINITY_DN730    177 TTCATACATAAAAAAAAGAAATTAACAGGTGTAACAAATTGACAACAACA    226
                     ||||||||||||||||||||||||||||||||||||||||||||||||||
TRINITY_DN273    234 TTCATACATAAAAAAAAGAAATTAACAGGTGTAACAAATTGACAACAACA    283

TRINITY_DN730    227 AAAAACAAAAATGCCTCTTGGAAGTTATTCTATGGGTCCACAATTTTCCG    276
                     ||||||||||||||||||||||||||||||||||||||||||||||||||
TRINITY_DN273    284 AAAAACAAAAATGCCTCTTGGAAGTTATTCTATGGGTCCACAATTTTCCG    333

TRINITY_DN730    277 CTCTTATGAATGGCTCTGTTGTTGATAAGGTGACGCCTGATATGGCGCAC    326
                     ||||||||||||||||||||||||||||||||||||||||||||||||||
TRINITY_DN273    334 CTCTTATGAATGGCTCTGTTGTTGATAAGGTGACGCCTGATATGGCGCAC    383

TRINITY_DN730    327 TTAATACAGCCATATTGGAATCAATTTCCCGCTATGGATCCTATGTGGAA    376
                     ||||||||||||||||||||||||||||||||||||||||||||||||||
TRINITY_DN273    384 TTAATACAGCCATATTGGAATCAATTTCCCGCTATGGATCCTATGTGGAA    433

TRINITY_DN730    377 TAAAATTTTAACAGCTTACATGATTTTAATTGGCTTGATATCGTGGTGTG    426
                     ||||||||||||||||||||||||||||||||||||||||||||||||||
TRINITY_DN273    434 TAAAATTTTAACAGCTTACATGATTTTAATTGGCTTGATATCGTGGTGTG    483

TRINITY_DN730    427 GCAATGGTGTCGTCATTTATATATTCACTACAACAAAATCCCTGCGTACA    476
                     ||||||||||||||||||||||||||||||||||||||||||||||||||
TRINITY_DN273    484 GCAATGGTGTCGTCATTTATATATTCACTACAACAAAATCCCTGCGTACA    533

TRINITY_DN730    477 CCTGCCAATCTGTTAGTTATTAATTTGGCATTATCTGATTTTGGTATGAT    526
                     ||||||||||||||||||||||||||||||||||||||||||||||||||
TRINITY_DN273    534 CCTGCCAATCTGTTAGTTATTAATTTGGCATTATCTGATTTTGGTATGAT    583

TRINITY_DN730    527 GGTAGTTAACACGCCCATGATGGGTACAAATTTATTCTTTGAAACGTGGA    576
                     ||||||||||||||||||||||||||||||||||||||||||||||||||
TRINITY_DN273    584 GGTAGTTAACACGCCCATGATGGGTACAAATTTATTCTTTGAAACGTGGA    633

TRINITY_DN730    577 TATGGGGACCAGCCGGTTGCGATGCATATGCAGCGTTAGGTTCAGCTTTT    626
                     ||||||||||||||||.|||||||||||||||||||||||||||||||||
TRINITY_DN273    634 TATGGGGACCAGCCGGCTGCGATGCATATGCAGCGTTAGGTTCAGCTTTT    683

TRINITY_DN730    627 GGTTGCGGTTCAATATGGTCCATGACTATGATTGCCTTAGATCGTTATAA    676
                     ||||||||||||||||||||||||||||||||||||||||||||||||||
TRINITY_DN273    684 GGTTGCGGTTCAATATGGTCCATGACTATGATTGCCTTAGATCGTTATAA    733
```

```
TRINITY_DN730     677  TGTTATAGTATTGGGCATGTCTGGACGTCCCATGACAATTAAGTTAGCTT    726
                       ||||||||||||||||||||||||||||||||||||||||||||||||||
TRINITY_DN273     734  TGTTATAGTATTGGGCATGTCTGGACGTCCCATGACAATTAAGTTAGCTT    783

TRINITY_DN730     727  TAATGAAGATTGCCTTCATTTGGGCTATGGCCAGCATTTGGACATTATCT    776
                       ||||||||||||||||||||||||||||||||||||||||||||||||||
TRINITY_DN273     784  TAATGAAGATTGCCTTCATTTGGGCTATGGCCAGCATTTGGACATTATCT    833

TRINITY_DN730     777  CCTATGTTCGGATGGAGTAGATATATTCCCGAAGGTAATTTAACCTCATG    826
                       ||||||||||||||||||||||||||||||||||||||||||||||||||
TRINITY_DN273     834  CCTATGTTCGGATGGAGTAGATATATTCCCGAAGGTAATTTAACCTCATG    883

TRINITY_DN730     827  TGGCATTGATTATTTGGGTCGTGAATGGAATGGTCGCAGCTATTTAATAT    876
                       ||||||||||||||||||||||||||||||||||||||||||||||||||
TRINITY_DN273     884  TGGCATTGATTATTTGGGTCGTGAATGGAATGGTCGCAGCTATTTAATAT    933

TRINITY_DN730     877  TGTATACAATCTTTGTATACTATATACCTCTATTTTTAATATGCTATTCA    926
                       ||||||||||||||||||||||||||||||||||||||||||||||||||
TRINITY_DN273     934  TGTATACAATCTTTGTATACTATATACCTCTATTTTTAATATGCTATTCA    983

TRINITY_DN730     927  TATTGGTTTATCATTGCCGCTGTATCGGCTCATGAGAAGGCTATGCGCGA    976
                       ||||||||||||||||||||||||||||||||||||||||||||||||||
TRINITY_DN273     984  TATTGGTTTATCATTGCCGCTGTATCGGCTCATGAGAAGGCTATGCGCGA   1033

TRINITY_DN730     977  ACAAGCCAAGAAAATGAATGTGAAATCATTGCGATCTTCAGAGGATGCTG   1026
                       ||||||||||||||||||||||||||||||||||||||||||||||||||
TRINITY_DN273    1034  ACAAGCCAAGAAAATGAATGTGAAATCATTGCGATCTTCAGAGGATGCTG   1083

TRINITY_DN730    1027  AAAAGAGTGCTGAAGGCAAATTAGCTAAGGTTGCCTTAGTCACTATATCA   1076
                       ||||||||||||||||||||||||||||||||||||||||||||||||||
TRINITY_DN273    1084  AAAAGAGTGCTGAAGGCAAATTAGCTAAGGTTGCCTTAGTCACTATATCA   1133

TRINITY_DN730    1077  TTGTGGTTCATGGCATGGACACCGTACACCATCATCAATATGGCTGGCTT   1126
                       ||||||||||||||||||||||||||||||||||||||||||||||||||
TRINITY_DN273    1134  TTGTGGTTCATGGCATGGACACCGTACACCATCATCAATATGGCTGGCTT   1183

TRINITY_DN730    1127  ATTCAAATTTGAAGGTCTCACCCCATTAAATACCATTTGGGGTGCTTGCT   1176
                       ||||||||||||||||||||||||||||||.|||||||||||||||||||
TRINITY_DN273    1184  ATTCAAATTTGAAGGTCTCACCCCATTAAACACCATTTGGGGTGCTTGCT   1233

TRINITY_DN730    1177  TTGCTAAATCAGCCGCTTGCTACAATCCTATTGTATACGGTATCAGCCAC   1226
                       ||||||||||||||||||||||||||||||||||||||||||||||||||
TRINITY_DN273    1234  TTGCTAAATCAGCCGCTTGCTACAATCCTATTGTATACGGTATCAGCCAC   1283

TRINITY_DN730    1227  CCTAAGTACCGGATTGCATTGAAAGAAAAATGTCCATGCTGTGTCTTTGG   1276
                       ||||||||||||||||||||||||||||||||||||||||||||||||||
TRINITY_DN273    1284  CCTAAGTACCGGATTGCATTGAAAGAAAAATGTCCATGCTGTGTCTTTGG   1333

TRINITY_DN730    1277  CAAAGTTGACGATGGTAAATCAGGTAGTGATGCTACCTCACAGGTTACTG   1326
                       ||||||||||||||||||||||||||||||||||||||||||||||||||
TRINITY_DN273    1334  CAAAGTTGACGATGGTAAATCAGGTAGTGATGCTACCTCACAGGTTACTG   1383

TRINITY_DN730    1327  CCAGTGAAGCAGAATCAAAGGCATAAACTTTTTCATAAATCGCACGCGTT   1376
                       ||||||||||||||||||||||||||||||||||||||||||||||||||
TRINITY_DN273    1384  CCAGTGAAGCAGAATCAAAGGCATAAACTTTTTCATAAATCGCACGCGTT   1433

TRINITY_DN730    1377  GATTTAGCATCAT-------------------------------------   1389
                       |||||||||||||
TRINITY_DN273    1434  GATTTAGCATCATCATTAAGTACCACAACAGCTGGACTAACACATAAACC   1483

TRINITY_DN730    1390  --------------------------------------------------   1389

TRINITY_DN273    1484  GATTAAACCCAACGAAAAAGCAGCAATAAATAAAATTATGTGATCTTAAA   1533

TRINITY_DN730    1390  --------------------------------------------------   1389

TRINITY_DN273    1534  ACTAATATCATAACAGTTCAATGCATGCTTGCTTACAAGATTAATTAAAC   1583

TRINITY_DN730    1390  --------------------------------------------------   1389

TRINITY_DN273    1584  CAAAGAAACGATAATAAAAATAATTTTATGCAAAGTCAATAAAGACCAAA   1633

TRINITY_DN730    1390  --------------------------------------------------   1389

TRINITY_DN273    1634  ATAAAGCAAATTCAGTAAGAAAAAAAAAAACAAACCAGTAAAAACATTAA   1683
```

Journal of Experimental Biology • Supplementary information

```
TRINITY_DN730    1390 --------------------------------------------------    1389

TRINITY_DN273    1684 ATTTGGTTGAGACCAAATCCCACCATTTAATGTTTCGTTGTATAGATGGT    1733

TRINITY_DN730    1390 --------------------------------------------------    1389

TRINITY_DN273    1734 GCAACAGCAACACCTTTATATATATGCGATAAAGTTGCGTACAAAAACCT    1783

TRINITY_DN730    1390 --------------------------------------------------    1389

TRINITY_DN273    1784 TAAAAAGGAGACGTAAAAATGGATGTTTTGTAACTTCTGGTAGACGTGGT    1833

TRINITY_DN730    1390 --------------------------------------------------    1389

TRINITY_DN273    1834 TGATTCCAATTTTTTTTACGCAGATAATATGAGGCAATATATATGATTTA    1883

TRINITY_DN730    1390 --------------------------------------------------    1389

TRINITY_DN273    1884 TATTCTTGCAAAATTTCAACCTTTTGTCTTTTGTAGTTTTTATGTAATGA    1933

TRINITY_DN730    1390 --------------------------------------------------    1389

TRINITY_DN273    1934 ATAAGAAACTTGTAAAAAAAAGTAAATATTTGACTTTATACAATTTGTGG    1983

TRINITY_DN730    1390 --------------------------------------------------    1389

TRINITY_DN273    1984 TAGACGCGCATGGCCAATTTAGATTTACTTTTTAAGCAGATATTATTAAA    2033

TRINITY_DN730    1390 --------------------------------------------------    1389

TRINITY_DN273    2034 CAATAATTTTTAAAGACGTGCGAAATTTCAACCGTACATCTCTTGTACAT    2083

TRINITY_DN730    1390 --------------------------------------------------    1389

TRINITY_DN273    2084 TTTTAGTAATAAGTAGAAAATTTATAAAAAGGCTATAATTGAGCACTATA    2133

TRINITY_DN730    1390 --------------------------------------------------    1389

TRINITY_DN273    2134 GAACTAGGCGATGGGCGTGGCTGATTTGAACAATTTTTGGATAGGAATTT    2183

TRINITY_DN730    1390 --------------------------------------------------    1389

TRINITY_DN273    2184 AATACTAGGTATCTTAAATATTTGCAAAATTTCAGCTTTGAGCAAAAGTC    2233

TRINITY_DN730    1390 --------------------------------------------------    1389

TRINITY_DN273    2234 CATAAATAATTATTTAATAAATAGTGATGTTATTACTTTACATGGCGGCT    2283

TRINITY_DN730    1390 --------------------------------------------------    1389

TRINITY_DN273    2284 ATTCCCTTCGACTGATTTGGCCCATTATGTATGCTTTAATTTCCTTAATT    2333

TRINITY_DN730    1390 --------------------------------------------------    1389

TRINITY_DN273    2334 ATGGAATGTTCGTCGTGAATAAACAAGGCTTTCAAACATGTTATATTGCC    2383

TRINITY_DN730    1390 --------------------------------------------------    1389

TRINITY_DN273    2384 ATTTGGACTTATCCTTACCCTTAAATATTTTCAGTATTAATGTACTTACA    2433

TRINITY_DN730    1390 --------------------------------------------------    1389

TRINITY_DN273    2434 AGATGTTACATCGAATTCGATATCTAATTTTTTGCGACCAGAACTAATAC    2483

TRINITY_DN730    1390 --------------------------------------------------    1389

TRINITY_DN273    2484 TAATTTAAAGCTATAACTAATTATGATTATGTGCAAATTTTGAGGGGTCT    2533

TRINITY_DN730    1390 --------------------------------------------------    1389

TRINITY_DN273    2534 CCGAAATTTACTTTACAAAATAACTGTACCTAAATTTTCAAGGATACAAC    2583

TRINITY_DN730    1390 --------------------------------------------------    1389

TRINITY_DN273    2584 GTTAATATTATAGTGACGAAATCGTATTCACTTTTGTTTATCATTCACAT    2633

TRINITY_DN730    1390 --------------------------------------------------    1389
```

Journal of Experimental Biology • Supplementary information

```
TRINITY_DN273   2634 CTTCAAATTGATTGCCAGGAACACACCAATTAACTTCAATTTTCTTATTA   2683

TRINITY_DN730   1390 --------------------------------------------------   1389

TRINITY_DN273   2684 TTTTTTGTATATTTATATTTATTAAATAGCATGTTCTTTTCAACTTAATG   2733

TRINITY_DN730   1390 --------------------------------------------------   1389

TRINITY_DN273   2734 CAATTACACTAAATTTTACACAAAAAGAAAATTTACAACAATTACCATAA   2783

TRINITY_DN730   1390 --------------------------------------------------   1389

TRINITY_DN273   2784 TCTCCTTATATTTTATATTAAGAGAGTTGGCACATCTTGATTGTATATTA   2833

TRINITY_DN730   1390 -----------------------------   1389

TRINITY_DN273   2834 GACTTAATAAGGAAGAAAAATTTATTCAC   2862
```

Fig. S3. Pairwise alignment of putative Rh1 opsin transcripts using EMBOSS needle (Rice et al., 2000), implemented using the EMBL-EBI job dispatcher (Madeira et al., 2024).

Journal of Experimental Biology • Supplementary information

```
#=======================================
#
# Aligned_sequences: 2
# 1: TRINITY_DN1081_c0_g2_i1-s4_head
# 2: TRINITY_DN1886_c0_g3_i2-w3_head
# Matrix: EBLOSUM62
# Gap_penalty: 10.0
# Extend_penalty: 0.5
#
# Length: 1614
# Identity:    1527/1614 (94.6%)
# Similarity:  1527/1614 (94.6%)
# Gaps:          87/1614 ( 5.4%)
# Score: 8253.0
#
#
#=======================================

TRINITY_DN108      1 GAATAACATTAGCTGAATAGAATTGTGCAAGTGAAATATTATAAAATCTA     50
                     ||||||||||||||||||||||||||||||||||||||||||||||||||
TRINITY_DN188      1 GAATAACATTAGCTGAATAGAATTGTGCAAGTGAAATATTATAAAATCTA     50

TRINITY_DN108     51 AATTGATTGGAACATTGGTACAAGATAGACGAAGGGGGAGTCGTTCATAG    100
                     ||||||||||||||||||||||||||||||||||||||||||||||||||
TRINITY_DN188     51 AATTGATTGGAACATTGGTACAAGATAGACGAAGGGGGAGTCGTTCATAG    100

TRINITY_DN108    101 AGAACTTTCTCTCGAAATTATTCCTATCGTGTTATCAGAAATAAATAAAT    150
                     ||||||||||||||||||||||||||||||||||||||||||||||||||
TRINITY_DN188    101 AGAACTTTCTCTCGAAATTATTCCTATCGTGTTATCAGAAATAAATAAAT    150

TRINITY_DN108    151 GTGATATCAGTGAGGGGAGAGTGCAAGAGGTGTAAAAAGAAATCTGATAT    200
                     ||||||||||||||||||||||||||||||||||||||||||||||||||
TRINITY_DN188    151 GTGATATCAGTGAGGGGAGAGTGCAAGAGGTGTAAAAAGAAATCTGATAT    200

TRINITY_DN108    201 AATGGCAGATTTTATGACACCAAAATTTCTACGTCAAATTAGCAATGGCT    250
                     ||||||||||||||||||||||||||||||||||||||||||||||||||
TRINITY_DN188    201 AATGGCAGATTTTATGACACCAAAATTTCTACGTCAAATTAGCAATGGCT    250

TRINITY_DN108    251 CCGTATTAGATAGA------------------------------------    264
                     ||||||||||||||
TRINITY_DN188    251 CCGTATTAGATAGAGTTTAGTATTCATGTTGTGAGATTTGCAAATAATTC    300

TRINITY_DN108    265 --------------------------------------------------    264

TRINITY_DN188    301 ATAATAAATTTGTGTATTTGCCTTGATATTATGTAATGTTTTTATAATCA    350

TRINITY_DN108    265 -GTTACACCGGATATGGTACATTTGGTTAATCCATATTGGGCTAGATTTC    313
                      |||||||||||||||||||||||||||||||||||||||||||||||||
TRINITY_DN188    351 GGTTACACCGGATATGGTACATTTGGTTAATCCATATTGGGCTAGATTTC    400

TRINITY_DN108    314 CACCCATGGAAACTTATATGAATCATACGTTGGCTTTATTTACTGGCATC    363
                     ||||||||||||||||||||||||||||||||||||||||||||||||||
TRINITY_DN188    401 CACCCATGGAAACTTATATGAATCATACGTTGGCTTTATTTACTGGCATC    450

TRINITY_DN108    364 ATAATGATAATATCATTATGTGGCAATGGCATGGTTGTATTCATATTCGG    413
                     ||||||||||||||||||||||||||||||||||||||||||||||||||
TRINITY_DN188    451 ATAATGATAATATCATTATGTGGCAATGGCATGGTTGTATTCATATTCGG    500

TRINITY_DN108    414 TAGTACAAAATCGCTGCGTACTCCCGCAAATCTATTGATCTTAAATTTGG    463
                     ||||||||||||||||||||||||||||||||||||||||||||||||||
TRINITY_DN188    501 TAGTACAAAATCGCTGCGTACTCCCGCAAATCTATTGATCTTAAATTTGG    550

TRINITY_DN108    464 CCTTTTCCGATTTTTGTATGATGGCATCACAAGCCCCAATTATGATAATT    513
                     ||||||||||||||||||||||||||||||||||||||||||||||||||
TRINITY_DN188    551 CCTTTTCCGATTTTTGTATGATGGCATCACAAGCCCCAATTATGATAATT    600

TRINITY_DN108    514 AATTTCTATTTCGAAACATGGATATTGGGACCATTATGGTGTGATATATA    563
                     ||||||||||||||||||||||||||||||||||||||||||||||||||
TRINITY_DN188    601 AATTTCTATTTCGAAACATGGATATTGGGACCATTATGGTGTGATATATA    650

TRINITY_DN108    564 TGCTATATGCGGTTCAATGTTTGGCTGTATTTCCATATGGACCATGTGCA    613
                     ||||||||||||||||||||||||||||||||||||||||||||||||||
TRINITY_DN188    651 TGCTATATGCGGTTCAATGTTTGGCTGTATTTCCATATGGACCATGTGCA    700

TRINITY_DN108    614 TGATAGCATTAGATCGTTATAATGTTATTGTTCGTGGCATGAACGGCCAA    663
                     ||||||||||||||||||||||||||||||||||||||||||||||||||
TRINITY_DN188    701 TGATAGCATTAGATCGTTATAATGTTATTGTTCGTGGCATGAACGGCCAA    750
```

Journal of Experimental Biology • Supplementary information

```
TRINITY_DN108     664 CCGATGACAGTTAAATTGGCTGTAATGAAAATTTTATTTATATGGTCTAT    713
                      ||||||||||||||||||||||||||||||||||||||||||||||||||
TRINITY_DN188     751 CCGATGACAGTTAAATTGGCTGTAATGAAAATTTTATTTATATGGTCTAT    800

TRINITY_DN108     714 AGCAACATTTTGGACTTTAATGCCAATGATTGGCTGGAATAATTATGTAC    763
                      ||||||||||||||||||||||||||||||||||||||||||||||||||
TRINITY_DN188     801 AGCAACATTTTGGACTTTAATGCCAATGATTGGCTGGAATAATTATGTAC    850

TRINITY_DN108     764 CTGAAGGCAATTTAACGGCCTGCTCTTTAGATTATTTAACACGTGATTGG    813
                      ||||||||||||||||||||||||||||||||||||||||||||||||||
TRINITY_DN188     851 CTGAAGGCAATTTAACGGCCTGCTCTTTAGATTATTTAACACGTGATTGG    900

TRINITY_DN108     814 AATCATCGTTCTTATCTGATTGTTTACTCTCTATTTGTTTATTATACACC    863
                      ||||||||||||||||||||||||||||||||||||||||||||||||||
TRINITY_DN188     901 AATCATCGTTCTTATCTGATTGTTTACTCTCTATTTGTTTATTATACACC    950

TRINITY_DN108     864 ATTATTTTTAATATGTTACTCTTATTGGTATATCATAGCGGCAGTGGCTG    913
                      ||||||||||||||||||||||||||||||||||||||||||||||||||
TRINITY_DN188     951 ATTATTTTTAATATGTTACTCTTATTGGTATATCATAGCGGCAGTGGCTG   1000

TRINITY_DN108     914 CTCATGAGAAGGCAATGCGTGAGCAGGCCAAAAAGATGAATGTAAAATCA    963
                      ||||||||||||||||||||||||||||||||||||||||||||||||||
TRINITY_DN188    1001 CTCATGAGAAGGCAATGCGTGAGCAGGCCAAAAAGATGAATGTAAAATCA   1050

TRINITY_DN108     964 TTGCGTTCATCGGAAGATTGTGAGAAATCGGCTGAAGCTAAATTAGCTAA   1013
                      ||||||||||||||||||||||||||||||||||||||||||||||||||
TRINITY_DN188    1051 TTGCGTTCATCGGAAGATTGTGAGAAATCGGCTGAAGCTAAATTAGCTAA   1100

TRINITY_DN108    1014 AGTGGCTTTAGTTACCATAACATTATGGTTTATGGCCTGGACTCCGTACT   1063
                      ||||||||||||||||||||||||||||||||||||||||||||||||||
TRINITY_DN188    1101 AGTGGCTTTAGTTACCATAACATTATGGTTTATGGCCTGGACTCCGTACT   1150

TRINITY_DN108    1064 TAATGATTTGCTATTTTGGTTTATTCAAAATTAATGGCTTAACACCGACA   1113
                      ||||||||||||||||||||||||||||||||||||||||||||||||||
TRINITY_DN188    1151 TAATGATTTGCTATTTTGGTTTATTCAAAATTAATGGCTTAACACCGACA   1200

TRINITY_DN108    1114 GCTACTGTATGGGGTGGTACATTCGCCAAAACAAGTGCCGTTTATAATCC   1163
                      ||||||||||||||||||||||||||||||||||||||||||||||||||
TRINITY_DN188    1201 GCTACTGTATGGGGTGGTACATTCGCCAAAACAAGTGCCGTTTATAATCC   1250

TRINITY_DN108    1164 TTTAGTCTATGGCATTAGTCATCCCAAATATCGTATGATCTTGAAGGAAA   1213
                      ||||||||||||||||||||||||||||||||||||||||||||||||||
TRINITY_DN188    1251 TTTAGTCTATGGCATTAGTCATCCCAAATATCGTATGATCTTGAAGGAAA   1300

TRINITY_DN108    1214 AGTGTCCATGGTTTGTTTGCGGCTCAACGGAGGAGACAAAACCTACCAAT   1263
                      ||||||||||||||||||||||||||||||||||||||||||||||||||
TRINITY_DN188    1301 AGTGTCCATGGTTTGTTTGCGGCTCAACGGAGGAGACAAAACCTACCAAT   1350

TRINITY_DN108    1264 AGTAGTAGTGATGCTCAAACGGCCGAAGGTGAATCTACAGCTTAATCTGT   1313
                      ||||||||||||||||||||||||||||||||||||||||||||||||||
TRINITY_DN188    1351 AGTAGTAGTGATGCTCAAACGGCCGAAGGTGAATCTACAGCTTAATCTGT   1400

TRINITY_DN108    1314 TTTAATTAATTTGCTAATGAATAAAGAGAAAAACGAATAAAAATCTTAAC   1363
                      ||||||||||||||||||||||||||||||||||||||||||||||||||
TRINITY_DN188    1401 TTTAATTAATTTGCTAATGAATAAAGAGAAAAACGAATAAAAATCTTAAC   1450

TRINITY_DN108    1364 AGAGCAATGTCATTTAAAAATGGCCAAAAATTATTTAAAAAAAAAAAAAA   1413
                      ||||||||||||||||||||||||||||||||||||||||||||||||||
TRINITY_DN188    1451 AGAGCAATGTCATTTAAAAATGGCCAAAAATTATTTAAAAAAAAAAAAAA   1500

TRINITY_DN108    1414 CAAAAGAAACCTAAACAAAAATTCAAAAAAAAAACTAAGATCCAATTAAG   1463
                      ||||||||||||||||||||||||||||||||||||||||||||||||||
TRINITY_DN188    1501 CAAAAGAAACCTAAACAAAAATTCAAAAAAAAAACTAAGATCCAATTAAG   1550

TRINITY_DN108    1464 AAAATTATTATGGAGAATTGTTAACAAAAAATAAAAATGGTACAAATAAA   1513
                      ||||||||||||||||||||||||||||||||||||||||||||||||||
TRINITY_DN188    1551 AAAATTATTATGGAGAATTGTTAACAAAAAATAAAAATGGTACAAATAAA   1600

TRINITY_DN108    1514 GATATTTAAGCATG   1527
                      ||||||||||||||
TRINITY_DN188    1601 GATATTTAAGCATG   1614
```

Fig. S4. Pairwise alignment of putative Rh2 opsin transcripts using EMBOSS needle (Rice et al., 2000), implemented using the EMBL-EBI job dispatcher (Madeira et al., 2024).

Journal of Experimental Biology • Supplementary information

Predicted protein sequences

The complete set of raw reads is available in the NCBI Sequence Read Archive associated with Bioproject PRJNA1391485, Biosamples SAMN54222331-SAMN54222346, and accession numbers SRR36528985-SRR36529000. cDNA and predicted protein sequences for all 11 putative opsin transcripts are provided in the following appendices.

Journal of Experimental Biology • Supplementary information

Fig. S5. The cDNA sequences for all 11 putative opsin transcripts.

```
>TRINITY_DN7305_c0_g1_i4-s2_head
ATTTTTTGTTTGAAATTTTTAAATTTCTGCAAACAATAAGAAAGTAATTGAGCAGACAAGTGCATAAAAAAAGGTATAAATTTGTTAACGTACA
TCAATAGGTTCCGCAGGTTTGAATATAACGGCGCCACGAATAGTCCTCATCGTAAATAATTTAGGAATTTTTCGTTAAACGTTTCATACATAAA
AAAAAGAAATTAACAGGTGTAACAAATTGACAACAACAAAAAACAAAAATGCCTCTTGGAAGTTATTCTATGGGTCCACAATTTTCCGCTCTTA
TGAATGGCTCTGTTGTTGATAAGGTGACGCCTGATATGGCGCACTTAATACAGCCATATTGGAATCAATTTCCCGCTATGGATCCTATGTGGAA
TAAAATTTTAACAGCTTACATGATTTTAATTGGCTTGATATCGTGGTGTGGCAATGGTGTCGTCATTTATATATTCACTACAACAAAATCCCTG
CGTACACCTGCCAATCTGTTAGTTATTAATTTGGCATTATCTGATTTTGGTATGATGGTAGTTAACACGCCCATGATGGGTACAAATTTATTCT
TTGAAACGTGGATATGGGGACCAGCCGGTTGCGATGCATATGCAGCGTTAGGTTCAGCTTTTGGTTGCGGTTCAATATGGTCCATGACTATGAT
TGCCTTAGATCGTTATAATGTTATAGTATTGGGCATGTCTGGACGTCCCATGACAATTAAGTTAGCTTTAATGAAGATTGCCTTCATTTGGGCT
ATGGCCAGCATTTGGACATTATCTCCTATGTTCGGATGGAGTAGATATATTCCCGAAGGTAATTTAACCTCATGTGGCATTGATTATTTGGGTC
GTGAATGGAATGGTCGCAGCTATTTAATATTGTATACAATCTTTGTATACTATATACCTCTATTTTTAATATGCTATTCATATTGGTTTATCAT
TGCCGCTGTATCGGCTCATGAGAAGGCTATGCGCGAACAAGCCAAGAAAATGAATGTGAAATCATTGCGATCTTCAGAGGATGCTGAAAAGAGT
GCTGAAGGCAAATTAGCTAAGGTTGCCTTAGTCACTATATCATTGTGGTTCATGGCATGGACACCGTACACCATCATCAATATGGCTGGCTTAT
TCAAATTTGAAGGTCTCACCCCATTAAATACCATTTGGGGTGCTTGCTTTGCTAAATCAGCCGCTTGCTACAATCCTATTGTATACGGTATCAG
CCACCCTAAGTACCGGATTGCATTGAAAGAAAAATGTCCATGCTGTGTCTTTGGCAAAGTTGACGATGGTAAATCAGGTAGTGATGCTACCTCA
CAGGTTACTGCCAGTGAAGCAGAATCAAAGGCATAAACTTTTTCATAAATCGCACGCGTTGATTTAGCATCAT
>TRINITY_DN2730_c0_g2_i6-s3_head
TTTTAAATTTCTGCAAACAATAAGAAAGTAATTGAGCAGACAAGTACATAAAAAAGGTATAAATTTGTTAACGTACATCAATAGGTTCCGCAGG
TTTGAATATAACGGCGCCACGAATAGTCCTCATCGGTAATTTCTCAAATAAAGTGCCAAAAATCAAATAAATGAAACTAGCAAATTGTGACGAA
ATTTAAACAATACAATAAATAATTTAGGAATTTTTGGTTAAACGTTTCATACATAAAAAAAAGAAATTAACAGGTGTAACAAATTGACAACAAC
AAAAAACAAAAATGCCTCTTGGAAGTTATTCTATGGGTCCACAATTTTCCGCTCTTATGAATGGCTCTGTTGTTGATAAGGTGACGCCTGATAT
GGCGCACTTAATACAGCCATATTGGAATCAATTTCCCGCTATGGATCCTATGTGGAATAAAATTTTAACAGCTTACATGATTTTAATTGGCTTG
ATATCGTGGTGTGGCAATGGTGTCGTCATTTATATATTCACTACAACAAAATCCCTGCGTACACCTGCCAATCTGTTAGTTATTAATTTGGCAT
TATCTGATTTTGGTATGATGGTAGTTAACACGCCCATGATGGGTACAAATTTATTCTTTGAAACGTGGATATGGGGACCAGCCGGCTGCGATGC
ATATGCAGCGTTAGGTTCAGCTTTTGGTTGCGGTTCAATATGGTCCATGACTATGATTGCCTTAGATCGTTATAATGTTATAGTATTGGGCATG
TCTGGACGTCCCATGACAATTAAGTTAGCTTTAATGAAGATTGCCTTCATTTGGGCTATGGCCAGCATTTGGACATTATCTCCTATGTTCGGAT
GGAGTAGATATATTCCCGAAGGTAATTTAACCTCATGTGGCATTGATTATTTGGGTCGTGAATGGAATGGTCGCAGCTATTTAATATTGTATAC
AATCTTTGTATACTATATACCTCTATTTTTAATATGCTATTCATATTGGTTTATCATTGCCGCTGTATCGGCTCATGAGAAGGCTATGCGCGAA
CAAGCCAAGAAAATGAATGTGAAATCATTGCGATCTTCAGAGGATGCTGAAAAGAGTGCTGAAGGCAAATTAGCTAAGGTTGCCTTAGTCACTA
TATCATTGTGGTTCATGGCATGGACACCGTACACCATCATCAATATGGCTGGCTTATTCAAATTTGAAGGTCTCACCCCATTAAACACCATTTG
GGGTGCTTGCTTTGCTAAATCAGCCGCTTGCTACAATCCTATTGTATACGGTATCAGCCACCCTAAGTACCGGATTGCATTGAAAGAAAAATGT
CCATGCTGTGTCTTTGGCAAAGTTGACGATGGTAAATCAGGTAGTGATGCTACCTCACAGGTTACTGCCAGTGAAGCAGAATCAAAGGCATAAA
CTTTTTCATAAATCGCACGCGTTGATTTAGCATCATCATTAAGTACCACAACAGCTGGACTAACACATAAACCGATTAAACCCAACGAAAAAGC
AGCAATAAATAAAATTATGTGATCTTAAAACTAATATCATAACAGTTCAATGCATGCTTGCTTACAAGATTAATTAAACCAAAGAAACGATAAT
AAAAATAATTTTATGCAAAGTCAATAAAGACCAAAATAAAGCAAATTCAGTAAGAAAAAAAAAAAACAAACCAGTAAAAACATTAAATTTGGTTG
AGACCAAATCCCACCATTTAATGTTTCGTTGTATAGATGGTGCAACAGCAACACCTTTATATATATGCGATAAAGTTGCGTACAAAAACCTTAA
AAAGGAGACGTAAAAATGGATGTTTTGTAACTTCTGGTAGACGTGGTTGATTCCAATTTTTTTTACGCAGATAATATGAGGCAATATATATGAT
TTATATTCTTGCAAAATTTCAACCTTTTGTCTTTTGTAGTTTTTATGTAATGAATAAGAAACTTGTAAAAAAAAGTAAATATTTGACTTTATAC
AATTTGTGGTAGACGCGCATGGCCAATTTAGATTTACTTTTTAAGCAGATATTATTAAACAATAATTTTTAAAGACGTGCGAAATTTCAACCGT
ACATCTCTTGTACATTTTTAGTAATAAGTAGAAAATTTATAAAAAGGCTATAATTGAGCACTATAGAACTAGGCGATGGGCGTGGCTGATTTGA
ACAATTTTTGGATAGGAATTTAATACTAGGTATCTTAAATATTTGCAAAATTTCAGCTTTGAGCAAAAGTCCATAAATAATTATTTAATAAATA
GTGATGTTATTACTTTACATGGCGGCTATTCCCTTCGACTGATTTGGCCCATTATGTATGCTTTAATTTCCTTAATTATGGAATGTTCGTCGTG
AATAAACAAGGCTTTCAAACATGTTATATTGCCATTTGGACTTATCCTTACCCTTAAATATTTTCAGTATTAATGTACTTACAAGATGTTACAT
CGAATTCGATATCTAATTTTTTGCGACCAGAACTAATACTAATTTAAAGCTATAACTAATTATGATTATGTGCAAATTTTGAGGGGTCTCCGAA
ATTTACTTTACAAAATAACTGTACCTAAATTTTCAAGGATACAACGTTAATATTATAGTGACGAAATCGTATTCACTTTTGTTTATCATTCACA
TCTTCAAATTGATTGCCAGGAACACACCAATTAACTTCAATTTTCTTATTATTTTTTGTATATTTATATTTATTAAATAGCATGTTCTTTTCAA
CTTAATGCAATTACACTAAATTTTACACAAAAAGAAAATTTACAACAATTACCATAATCTCCTTATATTTTATATTAAGAGAGTTGGCACATCT
TGATTGTATATTAGACTTAATAAGGAAGAAAAATTTATTCAC
>TRINITY_DN8493_c0_g1_i1-w2_head
CATTTTCCGATTTTTGCATGATGCTTACAAATGGTCCAATGATGGTTATTAATCTTTTCTTTGAAACTTGGGTACTTGGCCCATTAATGTGTGA
TATATACGCAGTCTGTGGTAGCATGTTTGGTTGTGTTTCTATTTGGTCAATGTGTATGGTTGCTTTCGATCGATACAATGTTATCGTAAAAGGT
ATCAATGGTACACCAATGACTATTAAATTAGCTATAATTAAAATTTTGGCAATATGGACAATGGCAACATTCTGGACTGTCGGGCCTATATTCG
GATGGAGCAGAAATGTACCAGAAGGTAACATGACATCATGTGGAATTGATTACTTGACAAGAGACTGGAATCCAAGATCTTATTTGATTTTTTA
TTCATTATTTGTGTACTACACTCCATTGTTCCTTATATGCTATTCTTACTGGTTTATTATTGCGGCCGTATCTGCTCACGAAAAAGCAATGCGC
GAACAAGCCAAAAAAATGAATGTCAAATCTCTCCGTTCATCCGAAGATGCTGACAAATCTGCTGAAGGAAAATTGGCTAAAGTAGCTTTAGTTA
CAATTTCTTTATGGTTCTGCGCATGGACACCATACATGGTTATCAACTACATGGGTCTTTTCAAATATGATAATATTACACCACTTATTACCAT
TTGGGGTGCTGTGTTTGCCAAAACTAGTGCCATATACAACCCAATTGTATATGGCATAAGCCATCCCAAATATCGTATTGCTCTTAAAGAGAAA
TGTCCATGCTGCGTATTCGGAAAAGTTGATGATGGTAAAGCAAGCGATGCGCAATCACAAGCAACAACAGAAGGAGGAGAAAGCAAGGCATAAA
TGTATAAAAAACAAAAATTGGCTAGTTTTCTACCGAGAATGTATAAATATATGAAAGTTATTTAGTGCATAACTTTGGCTTCGATCTTTACAT
TTTTTTATTTAAAGCAAAAAAAAACATTATTGGAAAATACCAT
>TRINITY_DN1081_c0_g2_i1-s4_head
GAATAACATTAGCTGAATAGAATTGTGCAAGTGAAATATTATAAAATCTAAATTGATTGGAACATTGGTACAAGATAGACGAAGGGGGAGTCGT
TCATAGAGAACTTTCTCTCGAAATTATTCCTATCGTGTTATCAGAAATAAATAAATGTGATATCAGTGAGGGGAGAGTGCAAGAGGTGTAAAAA
GAAATCTGATATAATGGCAGATTTTATGACACCAAAATTTCTACGTCAAATTAGCAATGGCTCCGTATTAGATAGAGTTACACCGGATATGGTA
CATTTGGTTAATCCATATTGGGCTAGATTTCCACCCATGGAAACTTATATGAATCATACGTTGGCTTTATTTACTGGCATCATAATGATAATAT
CATTATGTGGCAATGGCATGGTTGTATTCATATTCGGTAGTACAAAATCGCTGCGTACTCCCGCAAATCTATTGATCTTAAATTTGGCCTTTTC
CGATTTTTGTATGATGGCATCACAAGCCCCAATTATGATAATTAATTTCTATTTCGAAACATGGATATTGGGACCATTATGGTGTGATATATAT
GCTATATGCGGTTCAATGTTTGGCTGTATTTCCATATGGACCATGTGCATGATAGCATTAGATCGTTATAATGTTATTGTTCGTGGCATGAACG
GCCAACCGATGACAGTTAAATTGGCTGTAATGAAAATTTTATTTATATGGTCTATAGCAACATTTTGGACTTTAATGCCAATGATTGGCTGGAA
TAATTATGTACCTGAAGGCAATTTAACGGCCTGCTCTTTAGATTATTTAACACGTGATTGGAATCATCGTTCTTATCTGATTGTTTACTCTCTA
TTTGTTTATTATACACCATTATTTTTAATATGTTACTCTTATTGGTATATCATAGCGGCAGTGGCTGCTCATGAGAAGGCAATGCGTGAGCAGG
CCAAAAAGATGAATGTAAAATCATTGCGTTCATCGGAAGATTGTGAGAAATCGGCTGAAGCTAAATTAGCTAAAGTGGCTTTAGTTACCATAAC
ATTATGGTTTATGGCCTGGACTCCGTACTTAATGATTTGCTATTTTGGTTTATTCAAAATTAATGGCTTAACACCGACAGCTACTGTATGGGGT
```

Journal of Experimental Biology • Supplementary information

GGTACATTCGCCAAAACAAGTGCCGTTTATAATCCTTTAGTCTATGGCATTAGTCATCCCAAATATCGTATGATCTTGAAGGAAAAGTGTCCAT
GGTTTGTTTGCGGCTCAACGGAGGAGACAAAACCTACCAATAGTAGTAGTGATGCTCAAACGGCCGAAGGTGAATCTACAGCTTAATCTGTTTT
AATTAATTTGCTAATGAATAAAGAGAAAAACGAATAAAAATCTTAACAGAGCAATGTCATTTAAAAATGGCCAAAAATTATTTAAAAAAAAAAA
AAACAAAAGAAACCTAAACAAAAATTCAAAAAAAAAACTAAGATCCAATTAAGAAAATTATTATGGAGAATTGTTAACAAAAAATAAAAATGGT
ACAAATAAAGATATTTAAGCATG
>TRINITY_DN1886_c0_g3_i2-w3_head
GAATAACATTAGCTGAATAGAATTGTGCAAGTGAAATATTATAAAATCTAAATTGATTGGAACATTGGTACAAGATAGACGAAGGGGGAGTCGT
TCATAGAGAACTTTCTCTCGAAATTATTCCTATCGTGTTATCAGAAATAAATAAATGTGATATCAGTGAGGGGAGAGTGCAAGAGGTGTAAAAA
GAAATCTGATATAATGGCAGATTTTATGACACCAAAATTTCTACGTCAAATTAGCAATGGCTCCGTATTAGATAGAGTTTAGTATTCATGTTGT
GAGATTTGCAAATAATTCATAATAAATTTGTGTATTTGCCTTGATATTATGTAATGTTTTTATAATCAGGTTACACCGGATATGGTACATTTGG
TTAATCCATATTGGGCTAGATTTCCACCCATGGAAACTTATATGAATCATACGTTGGCTTTATTTACTGGCATCATAATGATAATATCATTATG
TGGCAATGGCATGGTTGTATTCATATTCGGTAGTACAAAATCGCTGCGTACTCCCGCAAATCTATTGATCTTAAATTTGGCCTTTTCCGATTTT
TGTATGATGGCATCACAAGCCCCAATTATGATAATTAATTTCTATTTCGAAACATGGATATTGGGACCATTATGGTGTGATATATATGCTATAT
GCGGTTCAATGTTTGGCTGTATTTCCATATGGACCATGTGCATGATAGCATTAGATCGTTATAATGTTATTGTTCGTGGCATGAACGGCCAACC
GATGACAGTTAAATTGGCTGTAATGAAAATTTTATTTATATGGTCTATAGCAACATTTTGGACTTTAATGCCAATGATTGGCTGGAATAATTAT
GTACCTGAAGGCAATTTAACGGCCTGCTCTTTAGATTATTTAACACGTGATTGGAATCATCGTTCTTATCTGATTGTTTACTCTCTATTTGTTT
ATTATACACCATTATTTTTAATATGTTACTCTTATTGGTATATCATAGCGGCAGTGGCTGCTCATGAGAAGGCAATGCGTGAGCAGGCCAAAAA
GATGAATGTAAAATCATTGCGTTCATCGGAAGATTGTGAGAAATCGGCTGAAGCTAAATTAGCTAAAGTGGCTTTAGTTACCATAACATTATGG
TTTATGGCCTGGACTCCGTACTTAATGATTTGCTATTTTGGTTTATTCAAAATTAATGGCTTAACACCGACAGCTACTGTATGGGGTGGTACAT
TCGCCAAAACAAGTGCCGTTTATAATCCTTTAGTCTATGGCATTAGTCATCCCAAATATCGTATGATCTTGAAGGAAAAGTGTCCATGGTTTGT
TTGCGGCTCAACGGAGGAGACAAAACCTACCAATAGTAGTAGTGATGCTCAAACGGCCGAAGGTGAATCTACAGCTTAATCTGTTTTAATTAAT
TTGCTAATGAATAAAGAGAAAAACGAATAAAAATCTTAACAGAGCAATGTCATTTAAAAATGGCCAAAAATTATTTAAAAAAAAAAAAAAACAAA
AGAAACCTAAACAAAAATTCAAAAAAAAAACTAAGATCCAATTAAGAAAATTATTATGGAGAATTGTTAACAAAAAATAAAAATGGTACAAATA
AAGATATTTAAGCATG
>TRINITY_DN2863_c0_g3_i3-s1_head
TGATTAATGCCGCGTGGGCATTTTTATATATGCACTTATATGTATAAATAAAAAAAAAAAATATATATATATACATACATATATATGCAAACGT
AAAAATATATATAATTAACTATACGTACACATATAGTTGTACTTATGCGTATAAATATAATTATTATAGAATTATGGATCCTAATCTTAAACCG
TTTACAATGGTAGACAATACTTCAAGTGTTATAAAGCCTTTAGCAAGAGTTGGAAGCGAAAGCCGATTATTAGGTTGGAATGTACCAGCAGAGG
AAATACGTCACATACCCGAACATTGGTTGCAATATGAAGAGCCACCAGCTTCATTGCATTATTTATTAGCAACTTTATATATATTTTTTACTAT
AATATCACTTCTAGGCAATGGTTTAGTTATTTGGATATTTACAGCTGCAAAATCTTTACGTACGCCTTCAAATATATTGGTGATCAATTTGGCC
GTATGTGATTTCTTTATGATGTTGAAAACACCAGTTTTCATTTACAACAGTTTTAATCGTGGTTTTGCTCTGGGCAATTTGGGCTGTCAAATTT
ACGGTATAATTGGTTCGTATACAGGGATTGGAGCATCAACTTCAAATGCCTTTATCGCTTATGATCGTTATAATGTTATAACGCGGCCATTGGA
AGGAAAAATGACTCATGGCAAAGCTATATTGATGATTTTATTTATATATTTATATGCAACACCATTTGTTGTGGCATGTTGCACTGAAAGTTGG
GGTAGATTTGTGCCAGAGGGATATTTAACATCTTGCACATTTGACTATCTAACCGATAATTTTGATACCCGATTGTTTGTCGGCACTATATTCT
TTTTTAGTTTCGTATGTCCCACATCGATGATTGTTTACTATTATAGTCAAATAGTTGGTCACGTTTTCAGCCATGAGAAGGCATTACGAGATCA
AGCGAAAAAAATGAATGTTGAATCATTACGTTCCAATGTTGATAAAAGTAAAGATACTGCGGAGATCCGTATAGCAAAAGCTGCTATCACTATT
TGTTTTCTATTTTTCATATCATGGACGCCCTATGGAGTTATGTCATTGATTGGAGCCTTTGGCGATAAAAGCCTATTAACACCTGGTGTAACCA
TGATACCAGCCTGCACATGCAAAATGGTTGCTTGCGTTGATCCTTTTGTATATGCAATAAGTCATCCAAAATACCGTTTAGAGTTACAAAAACG
TTGTCCTTGGTTAGCCATAAATGAAAAAGCTGCAGAAGTATCAGCAACGGCTTCTACAACGACTCAAGATCAAATTGCATTGCAAACCAACACT
TAAATCCATACTTGAATTCTTAAATCTAACAATGGAACCTTTGAATAATGATGTAAACGTGAAAAAAGGCACAACTTATATGTAACCAATGACT
ACGATTTTAATTTCATTGAAAATTATATTTATGGCTCCATAAAAATGGGGATTTGTTAGAAATAAATCCGATTAAAATTTCACTTTGTGTTGTA
GTATATTTTAAGTTTATTAATCTGAAATAAATAGAGAAATTATTGCAAGC
>TRINITY_DN2863_c0_g3_i2-s1_head
GCATTTTAACATGGCTTATCATTTTCACAAAATATATTTGGACTATTATTTTGTCCAAATTGATTATTTAATATTCATTTAATTACACTTTTAT
GTTCGAAGATAATTGCTTTTTTTTAGTGGGCACACTTTTCTTCTTTTAAGCATTTACAATATATTCTATAAATTAACGAAGTGAATTTGCTTCG
GCTCTACAAACTAAATTTTTTTAAATTTGTTATGACACTTTCACGATAATTTATATCACTTTTATTGTTTTCATAACATTTTATCTCTCGACAC
AATTCAATATGGCAGCCATGTTGTTCACAGTGCTCTTGATAAATTACTAATCAGTGTAGATTTTATATTAAAAATTTGTAGTCGTTAATTAATT
TAACAATGAAAACATAAGAATCAAATTAATGTTATTCGATTAAATAACATGCGCTATTCAACCTAAATTAATTTCCAAACTTAAATAGTGACTT
ACACTTTTTCGTCGATCTACGAAGAAATACACAAAAAGAAATGACAATATATTTATCCCTGCCATGTTATTTTTTACATGAAATAAAAAATCAA
CATTTTTCTCTTTCTATATGGTGTGCAACTTGTAATAAGCAACATTTCTCTGCTTTACCAATTTGTGCAGATATTTTTAATGCACAAGAAAAAG
AGTTGAGATGGGTCCAACTTTTACAAGCCGACTCAGAAAGAATTTCTAAATTGGTTTAAGTTTCTCGACTTTGATTGAAGTCGTTATATGAGAA
AAGAATTATACTTTCGAAATGTTTTGACGAAAATTGGCACGTCATTGTATTTTGATGTACTAAATCAGTCCATTTTTTTGGATCCCTCAACGTA
TTTACTTGACTGTTTAGTTACATTAAAGGGATATTTAACATCTTGCACATTTGACTATCTAACCGATAATTTTGATACCCGATTGTTTGTCGGC
ACTATATTCTTTTTTAGTTTCGTATGTCCCACATCGATGATTGTTTACTATTATAGTCAAATAGTTGGTCACGTTTTCAGCCATGAGAAGGCAT
TACGAGATCAAGCGAAAAAAATGAATGTTGAATCATTACGTTCCAATGTTGATAAAAGTAAAGATACTGCGGAGATCCGTATAGCAAAAGCTGC
TATCACTATTTGTTTTCTATTTTTCATATCATGGACGCCCTATGGAGTTATGTCATTGATTGGAGCCTTTGGCGATAAAAGCCTATTAACACCT
GGTGTAACCATGATACCAGCCTGCACATGCAAAATGGTTGCTTGCGTTGATCCTTTTGTATATGCAATAAGTCATCCAAAATACCGTTTAGAGT
TACAAAAACGTTGTCCTTGGTTAGCCATAAATGAAAAAGCTGCAGAAGTATCAGCAACGGCTTCTACAACGACTCAAGATCAAATTGCATTGCA
AACCAACACTTAAATCCATACTTGAATTCTTAAATCTAACAATGGAACCTTTGAATAATGATGTAAACGTGAAAAAAGGCACAACTTATATGTA
ACCAATGACTACGATTTTAATTTCATTGAAAATTATATTTATGGCTCCATAAAAATGGGGATTTGTTAGAAATAAATCCGATTAAAATTTCACT
TTGTGTTGTAGTATATTTTAAGTTTATTAATCTGAAATAAATAGAGAAATTATTGCAAGC
>TRINITY_DN7379_c0_g4_i3-s2_head
TGCAAACGTAAAAATATATATAATTAACTATACGTACACATATAGTTGTACTTATGCGTATAAATATAATTATTATAGAATTATGGATCCTAAT
CTTAAACCGTTTACAATGGTAGACAATACTTCAAGTGTTATAAAGCCTTTAGCAAGAGTTGGAAGCGAAAGCCGATTATTAGGTTGGAATGTAC
CAGCAGAGGAAATACGTCACATACCCGAACATTGGTTGCAATATGAAGAGCCACCAGCTTCATTGCATTATTTATTAGCAACTTTATATATATT
TTTTACTATAATATCACTTCTAGGCAATGGTTTAGTTATTTGGATATTTACAGCTGCAAAATCTTTACGTACGCCTTCAAATATATTGGTGATC
AATTTGGCCGTATGTGATTTCTTTATGATGTTGAAAACACCAGTTTTCATTTACAACAGTTTTAATCGTGGTTTTGCTCTGGGCAATTTGGGCT
GTCAAATTTACGGTATAATTGGTTCGTATACAGGGATTGGAGCATCAACTTCAAATGCCTTTATCGCTTATGATCGTTATAATGTTATAACGCG
GCCATTGGAAGGAAAAATGACTCATGGCAAAGCTATATTGATGATTTTATTTATATATTTATATGCAACACCATTTGTTGTGGCATGTTGCACT
GAAAGTTGGGGTAGATTTGTGCCAGGTAAATATAACCTTTACATAAACGTACAGTTTATATTAACAACATAAGGGTCAGTATCAAGTTATTATA
GGAATTATTTTCTTGTCTCTTTGGCAGTAATTTACAATAAAAATTAAGTAACGGTTACAGTATGCATTTTCAAATTCTGCCCCTATACCATAAA
AATGTATGTTATATTACGCTATACGAGTCGCCTTAGTGCTTTGTCATTTATACCGTAAATTATGAAAAATGAGTAACTTTTGAGGATTTTTAGT
AGACATTGTCGTCTCCATTACTGCACTTACACGTCTTTCGATTTAGAATTGATTTCTTAATTGGCTTGCAAATTGCCTGCATATTATGAAAAAT
TACAGTTATTTTGTAATCCTATTTTATTTTATTGATTTATTTATATATATCATTATTATCA
>TRINITY_DN1981_c1_g1_i1-w1_head
TTTTTTAAAATTTGTTATGACACTTTCACGATAATTTATATCACTTTTATTGTTTTCATAACATTTTATCTCTCGACACAATTCAATATGGCAG
CCATGTTGTTCACAGTGCTCTTGATAAATTACTAATCAGTGTAGATTTTATATTAAAAATTTGTAGTCGTTAATTAATTTAACAATGAAAACAT

Journal of Experimental Biology • Supplementary information

AAGAATCAAATTAATGTTATTCGATTAAATAACATGCGCTATTCAACCTAAATTAATTTCCAAACTTAAATAGTGACTTACACTTTTTCGTCGA
TCTACGAAGAAATACACAAAAAGAAATGACAATATATTTATCCCTGCCATGTTATTTTTTACATGAAATAAAAAATCAACATTTTTCTCTTTCT
ATATGGTGTGCAACTTGTAATAAGCAACATTTCTCTGCTTTACCAATTTGTGCAGATATTTTTAATGCACAAGAAAAAGAGTTGAGATGGGTCC
AACTTTTACAAGCCGACTCAGAAAGAATTTCTAAATTGGTTTAAGTTTCTCGACTTTGATTGAAGTCGTTATATGAGAAAAGAATTATACTTTC
GAAATGTTTTGACGAAAATTGGCACGTCATTGTATTTTGATGTACTAAATCAGTCCATTTTTTTGGATCCCTCAACGTATTTACTTGACTGTTT
AGTTACATTAAGTAAGGTGGTGAAAGACATTTAACAGTTGGCTCCGATTGACTAAAAAGTTATTGTTTTCCATTCGTTTACTTTCAGAGGGATA
TTTAACATCTTGCACATTTGACTATCTAACCGATAATTTTGATACCCGATTGTTTGTCGGCACTATATTCTTTTTTAGTTTCGTATGTCCCACA
TCGATGATTGTTTACTATTATAGTCAAATAGTTGGTCACGTTTTCAGCCATGAGAAGGCATTACGAGATCAAGCGAAAAAAATGAATGTTGAAT
CATTACGTTCCAATGTTGATAAAAGTAAAGATACTGCGGAGATCCGTATAGCAAAAGCTGCTATCACTATTTGTTTTCTATTTTTCATATCATG
GACGCCCTATGGAGTTATGTCATTGATTGGAGCCTTTGGCGATAAAAGCCTATTAACACCTGGTGTAACCATGATACCAGCCTGCACATGCAAA
ATGGTTGCTTGCGTTGATCCTTTTGTATATGCAATAAGTCATCCAAAATACCGTTTAGAGTTACAAAAACGTTGTCCTTGGTTAGCCATAAATG
AAAAAGCTGCAGAAGTATCAGCAACGGCTTCTACAACGACTCAAGATCAAATTGCATTGCAAACCAACACTTAAATCCATACTTGAATTCTTAA
ATCTAACAATGGAACCTTTGAATAATGATGTAAACGTGAAAAAAGGCACAACTTATATGTAACCAATGACTACGATTTTAATTTCATTGAAAAT
TATATTTATGGCTCCATAAAAATGGGGATTTGTTAGAAATAAATCCGATTAAAATTTCACTTTGTGTTGTAGTATATTTTAAGTTTATTAATCT
GAAATAAATAGAGAAATTATTGCAAGC
>TRINITY_DN1244_c0_g1_i1-w1_head
GTTTTTTTTATTGGGCATAAAATTAAATACGGAAAAACAGAAGAAAAAAAACGGTATTTACAATAAGAGAAAGTGACAGTAGGTGTAATTTGAA
ATTGGGCCAATGGCAAGGCGGCCCAAAATGGGTCCTGAAACCTACAACATTCATATAAATTACTCAAAGGATGCATCTGTGCCAAATGAATTGC
CTTATTTAGGTTGGAGTTATCCATATGAATATCAACATATGGTCCATCAACATTGGCGTTCATTTTCCGAGCCATCTCTCTACTATGAAATCGC
TTTTGTCATCTTTTTCGCAGTTCTACTATTAACCTCTCTGTTTGGCAATGGTTTAGTTATCTGGATTTTTTCAACAGCGAAAACATTACGGACC
CCATCAAATCTTTTGATTATCAATTTGGCAGCATTCGATTTAACTATGGCTCTTAATATGCCTCACTACTTGATCAATGCAGTTATGAGATATT
TTCCTGGTGGTGATTTAGCCTGTGATATATATGCTGTCTTGGGTGGCATTTCTGGCATGGGCGCGGCAATCACAAACGCCTTCATTGCTTATGA
TCGCTATAGAACAATCTCGAATCCACTTGATGGCCGTTTAAACTATAAACAAATAATCATATTGATTGCAATATCATGGCTATGGACTATACCG
TTTTCTGTGTTGCCATTTTTACATATCTGGGGCCGCTATATACCAGAGGGCTTTCTAACAAGTTGCTCTTTCGATTATTTAACGGATGATGATG
AGACACGATATTTTGTAAGGGCCATGTTCATATGGGCCTATTGTATACCAATGATCGTGATATGTATATATTATACAAAGCTATTTCTACATGT
CCGCCAACATGAGCAAATGTTGGCCGATCAAGCGAAAAAAATGAATGTCAAATCATTGATAGCGTATCCACATAATACCGGTTTAAGTACAGAA
TTACGTATAGCAAAAGCTGCCATGATTATATATTTAATGTATGTATTCGCTTGGACTCCATACGCCACAATTGCCCTGTTGGGCACTTTTGGCT
ACTCACATCTAATTACGCCATTCGCTTCAATGATACCGTGTTGTTGTGCCAAAATAGTATCATGTCTTGATCCTTGGATTTATGCGGCCAGCCA
TCCAAAATATCGTGCAGTTTTAGCAAGTCGTCTCCCCTTTTTGGGTATACAAGAAAAAGGTAATTCAATAACATCCTCAAATGACAATGGAAAT
ATTAATGGTGGTGGCTCTAATGATGGCGATGCTACAACTTTATCGGTGATTGCCTAAATATTTCCTAGAATGTAAACCATGAATCACTCTAATC
TTCTTTTTACGTATTTCAACCATGCATACAAATTTTTAAAGCCCTGCATCGCAACTAGAATTAAAATAAAATAATTATTAATTTTACTTAATTT
AAAAAGATTAAGGTAGAATGCAGTAACACTATGTAATAAGTCTTAAAAGACTTCAAAAATACAAAAAAAAAAATAACATAATTAGCAATAAGCCA
GAAAGGCTTGAGAAATTTATAACAAAAATCAAAAAAAAAAATTAATAAATAATGATGCAATAAAAATGATTTATTAATAAGAAGCATAAAAAGC
AGAGATTTTATATTGTTTAAATTTCTCTGTTAAATGAAATTAAACCAATTAACGGTAGAACGGAACTGTTTTAACAGATGCGAAAAATTTGTAG
TAAAATACTATAAATTGCCTAATACTGAAAATATCAACTGTGCTTATAAATTTGTTAATTATCATGTATATTGAATTTATGAAGAAGAAGAGCA
ATAAAGAAACTAA
>TRINITY_DN435_c0_g5_i1-w4_head
ACTTAATTGACAATTCCATTGAGTTATATTGCAAGTCAAATAAAGATTTGCAGGCAATAACCACCAGAAATTCCAAGAAATTTCAAAGTTTCAA
GGTGCTTTCTGCTATAGACAAATTTGTAATGAGAAAATTTTGAACAAAGAAAGATTCTTTTTCTTTTCAAAGAAAAATATTAATAATTTAAAAT
AATTTATGTGGCAGTGAAAGTATCCAGTTGGTGGTAACTATGATATCGCAAAAAATTGGCATTATTCAATATTTACATGGTGGCCCGAGTTTGG
GTTCTGGCCATAATATCTCATTAGCTGAGAGTGTACCGGCCGAAATCTATCATATGGTAGATCCCTATTGGTATCAATGGCCACCAATGGATCA
AATGTGGTTCGGTATTATCGCTTTTGTTATAACCATACTTGGTATAATGTCACTCTCAGGCAATTTCATTGTTATGTATATATTTACATCATCA
AAATCATTACGAACACCATCCAATATGTTTGTGGTAAATTTGGCATTTTCGGATTTTATGATGATGTTCACTATGTTTCCACCCGTTGTACTGA
ATAGTTTTTATGGTACATGGATATTAGGTCCATTATGGTGTGAACTCTATGGCTTATTTGGTTCATTATTCGGTTGTGTATCAATTTGGTCCAT
GACATTAATCGCTTATGATCGTTATTGTGTTATCGTGAAAGGGCTTTCAGCTAAACCATTAACTAGTTCCATCGCTGCATTACGTTTATTGGTC
GTTTGGATAATTTGCTTGAGTTGGGGTTTATTACCTATGTTTGGTTGGAATCGTTACGTGCCGGAAGGGAATATGACAGCATGTGGTACGGATT
ATTTTGCTAAAGATTGGTGGAATCGTTCCTACATAATTGTTTACTCACTCTGGGTCTATTTTACACCGCTATTAACCATCATATTTTCTTATTG
GCATATTTTAAAGGCTGTTAGAGCTCATGAGCAGGCGATGCGAGAACAAGCGAAAAAAATGAATGTATCATCACTACGTAATACGGAGACAGAT
AAGGGGAAATCGGTTGAAATTAAATTGGCCAAAGTAGCTTTAATCACCATAACATTATGGTTTATGGCATGGACACCGTATACAATTATCAATT
ATGCCGGTATATTCGAGTCCATGCATCTATCACCGTTAAGTACAATTTGTGGTTCAATTTTCGCTAAAGCAAATTCTATTTATAATCCAATTGT
CTATGGACTTAGTCATCCGAAATATAAACAAGTACTGCGAGAAAAGATGCCATGCTTGGTATGCGGCAAAGATGATAATAATGCAGACGTTCGT
ACACAAGCGACTGGTGAAGTTAGTGAATCGATAGCGTAAATCTTGTGTGCACTATGAAAGGCCACCTTTAATGATCAAATGACTTTTCTCTTCA
CGGCCTAATGATATGAAAATGCAACAATGACGTTTTAACAAGCTTTAAAAAAAAAGCCAAAAAAGACCAAAAAAAAAAAACAGAAAATCAAAAACA
AACTACAGAGGAGAATGAAATTTGTATACAAGTTTCTTAATTTAGAACAATGTGATGTATTGAGAATAATCCATAAGTAAAAAGCAAATAGCAA
TTGATATAAAATCAAACAAATTATGGTTGAAATTTTCCTTTTTTTTTTGTTTAAATAAGATGGAAAGAAAGAAAAACTCATTCAAAAAACATATT
CAGAAAGAATCAATTGTACCATAACTGCTCTAGATTGAAACATCTAATTTACCAATCGCGTCATATGAAAATTTATGTTATACCCTAAGAAACT
TTAAAGAATTCTCAGGCTTTCTCTTCAAAATAACATTAAGATTTTTAAAAAACTTTTAGAGCTAGTATCGCATTTATTCATCCATTTGTTTACT
TTTTATATTATAATTTTCAAAATACCTACAATTCATTAACTTGTTTACGTTTTTAGTTTATAGATCTAATTCCATTAAAAATAGCTTAAATAAG
ATTTGGCTATTTCGTAAATATCCATTTTAGAGGAAAATGCACACTTAATTATCATATCAAAAGTAGAACCACGCCTAAAACTATTTACTACAAA
ACTATAACGAATCTTAGAAATTTAAAAAAAAAGAAAAGTTACTGCATTTCACTTGTTTCGCTTTATGTTTGTTAGAGTGACGGGCATTTAAAGT
ATTTAAGAGACGATTTCAACAGTCTACTTGTTTTATTTACATATATTTTGAAAT

Fig. S6. The predicted protein sequences for all 11 putative opsin transcripts.

```
>TRINITY_DN7305_c0_g1_i4-s2_head|m.112 TRINITY_DN7305_c0_g1_i4-s2_head|g.112 type:complete
len:372 TRINITY_DN7305_c0_g1_i4-s2_head:237-1352(+)
MPLGSYSMGPQFSALMNGSVVDKVTPDMAHLIQPYWNQFPAMDPMWNKILTAYMILIGLISWCGNGVVIYIFTTTKSLRTPANLLVINLALSDF
GMMVVNTPMMGTNLFFETWIWGPAGCDAYAALGSAFGCGSIWSMTMIALDRYNVIVLGMSGRPMTIKLALMKIAFIWAMASIWTLSPMFGWSRY
IPEGNLTSCGIDYLGREWNGRSYLILYTIFVYYIPLFLICYSYWFIIAAVSAHEKAMREQAKKMNVKSLRSSEDAEKSAEGKLAKVALVTISLW
FMAWTPYTIINMAGLFKFEGLTPLNTIWGACFAKSAACYNPIVYGISHPKYRIALKEKCPCCVFGKVDDGKSGSDATSQVTASEAESKA*
>TRINITY_DN2730_c0_g2_i6-s3_head|m.34 TRINITY_DN2730_c0_g2_i6-s3_head|g.34 type:complete
len:372 TRINITY_DN2730_c0_g2_i6-s3_head:294-1409(+)
MPLGSYSMGPQFSALMNGSVVDKVTPDMAHLIQPYWNQFPAMDPMWNKILTAYMILIGLISWCGNGVVIYIFTTTKSLRTPANLLVINLALSDF
GMMVVNTPMMGTNLFFETWIWGPAGCDAYAALGSAFGCGSIWSMTMIALDRYNVIVLGMSGRPMTIKLALMKIAFIWAMASIWTLSPMFGWSRY
IPEGNLTSCGIDYLGREWNGRSYLILYTIFVYYIPLFLICYSYWFIIAAVSAHEKAMREQAKKMNVKSLRSSEDAEKSAEGKLAKVALVTISLW
FMAWTPYTIINMAGLFKFEGLTPLNTIWGACFAKSAACYNPIVYGISHPKYRIALKEKCPCCVFGKVDDGKSGSDATSQVTASEAESKA*
>TRINITY_DN8493_c0_g1_i1-w2_head|m.19 TRINITY_DN8493_c0_g1_i1-w2_head|g.19 type:5prime_partial
len:281 TRINITY_DN8493_c0_g1_i1-w2_head:3-845(+)
FSDFCMMLTNGPMMVINLFFETWVLGPLMCDIYAVCGSMFGCVSIWSMCMVAFDRYNVIVKGINGTPMTIKLAIIKILAIWTMATFWTVGPIFG
WSRNVPEGNMTSCGIDYLTRDWNPRSYLIFYSLFVYYTPLFLICYSYWFIIAAVSAHEKAMREQAKKMNVKSLRSSEDADKSAEGKLAKVALVT
ISLWFCAWTPYMVINYMGLFKYDNITPLITIWGAVFAKTSAIYNPIVYGISHPKYRIALKEKCPCCVFGKVDDGKASDAQSQATTEGGESKA*
>TRINITY_DN8493_c0_g1_i1-w2_head|m.20 TRINITY_DN8493_c0_g1_i1-w2_head|g.20 type:3prime_partial
len:105 TRINITY_DN8493_c0_g1_i1-w2_head:313-2(-)
MLPSGTFLLHPNIGPTVQNVAIVHIAKILIIANLIVIGVPLIPFTITLYRSKATIHIDQIETQPNMLPQTAYISHINGPSTQVSKKRLITIIGP
FVSIMQKSEN
>TRINITY_DN1081_c0_g2_i1-s4_head|m.144 TRINITY_DN1081_c0_g2_i1-s4_head|g.144 type:complete
len:369 TRINITY_DN1081_c0_g2_i1-s4_head:202-1308(+)
MADFMTPKFLRQISNGSVLDRVTPDMVHLVNPYWARFPPMETYMNHTLALFTGIIMIISLCGNGMVVFIFGSTKSLRTPANLLILNLAFSDFCM
MASQAPIMIINFYFETWILGPLWCDIYAICGSMFGCISIWTMCMIALDRYNVIVRGMNGQPMTVKLAVMKILFIWSIATFWTLMPMIGWNNYVP
EGNLTACSLDYLTRDWNHRSYLIVYSLFVYYTPLFLICYSYWYIIAAVAAHEKAMREQAKKMNVKSLRSSEDCEKSAEAKLAKVALVTITLWFM
AWTPYLMICYFGLFKINGLTPTATVWGGTFAKTSAVYNPLVYGISHPKYRMILKEKCPWFVCGSTEETKPTNSSSDAQTAEGESTA*
>TRINITY_DN1886_c0_g3_i2-w3_head|m.178 TRINITY_DN1886_c0_g3_i2-w3_head|g.178 type:complete
len:344 TRINITY_DN1886_c0_g3_i2-w3_head:364-1395(+)
MVHLVNPYWARFPPMETYMNHTLALFTGIIMIISLCGNGMVVFIFGSTKSLRTPANLLILNLAFSDFCMMASQAPIMIINFYFETWILGPLWCD
IYAICGSMFGCISIWTMCMIALDRYNVIVRGMNGQPMTVKLAVMKILFIWSIATFWTLMPMIGWNNYVPEGNLTACSLDYLTRDWNHRSYLIVY
SLFVYYTPLFLICYSYWYIIAAVAAHEKAMREQAKKMNVKSLRSSEDCEKSAEAKLAKVALVTITLWFMAWTPYLMICYFGLFKINGLTPTATV
WGGTFAKTSAVYNPLVYGISHPKYRMILKEKCPWFVCGSTEETKPTNSSSDAQTAEGESTA*
>TRINITY_DN2863_c0_g3_i3-s1_head|m.69 TRINITY_DN2863_c0_g3_i3-s1_head|g.69 type:complete
len:384 TRINITY_DN2863_c0_g3_i3-s1_head:168-1319(+)
MDPNLKPFTMVDNTSSVIKPLARVGSESRLLGWNVPAEEIRHIPEHWLQYEEPPASLHYLLATLYIFFTIISLLGNGLVIWIFTAAKSLRTPSN
ILVINLAVCDFFMMLKTPVFIYNSFNRGFALGNLGCQIYGIIGSYTGIGASTSNAFIAYDRYNVITRPLEGKMTHGKAILMILFIYLYATPFVV
ACCTESWGRFVPEGYLTSCTFDYLTDNFDTRLFVGTIFFFSFVCPTSMIVYYYSQIVGHVFSHEKALRDQAKKMNVESLRSNVDKSKDTAEIRI
AKAAITICFLFFISWTPYGVMSLIGAFGDKSLLTPGVTMIPACTCKMVACVDPFVYAISHPKYRLELQKRCPWLAINEKAAEVSATASTTTQDQ
IALQTNT*
>TRINITY_DN2863_c0_g3_i2-s1_head|m.68 TRINITY_DN2863_c0_g3_i2-s1_head|g.68 type:complete
len:226 TRINITY_DN2863_c0_g3_i2-s1_head:746-1423(+)
MRKELYFRNVLTKIGTSLYFDVLNQSIFLDPSTYLLDCLVTLKGYLTSCTFDYLTDNFDTRLFVGTIFFFSFVCPTSMIVYYYSQIVGHVFSHE
KALRDQAKKMNVESLRSNVDKSKDTAEIRIAKAAITICFLFFISWTPYGVMSLIGAFGDKSLLTPGVTMIPACTCKMVACVDPFVYAISHPKYR
LELQKRCPWLAINEKAAEVSATASTTTQDQIALQTNT*
>TRINITY_DN7379_c0_g4_i3-s2_head|m.113 TRINITY_DN7379_c0_g4_i3-s2_head|g.113
type:5prime_partial len:243 TRINITY_DN7379_c0_g4_i3-s2_head:2-730(+)
ANVKIYIINYTYTYSCTYAYKYNYYRIMDPNLKPFTMVDNTSSVIKPLARVGSESRLLGWNVPAEEIRHIPEHWLQYEEPPASLHYLLATLYIF
FTIISLLGNGLVIWIFTAAKSLRTPSNILVINLAVCDFFMMLKTPVFIYNSFNRGFALGNLGCQIYGIIGSYTGIGASTSNAFIAYDRYNVITR
PLEGKMTHGKAILMILFIYLYATPFVVACCTESWGRFVPGKYNLYINVQFILTT*
>TRINITY_DN1981_c1_g1_i1-w1_head|m.143 TRINITY_DN1981_c1_g1_i1-w1_head|g.143 type:complete
len:149 TRINITY_DN1981_c1_g1_i1-w1_head:850-1296(+)
MIVYYYSQIVGHVFSHEKALRDQAKKMNVESLRSNVDKSKDTAEIRIAKAAITICFLFFISWTPYGVMSLIGAFGDKSLLTPGVTMIPACTCKM
VACVDPFVYAISHPKYRLELQKRCPWLAINEKAAEVSATASTTTQDQIALQTNT*
>TRINITY_DN1244_c0_g1_i1-w1_head|m.137 TRINITY_DN1244_c0_g1_i1-w1_head|g.137 type:complete
len:392 TRINITY_DN1244_c0_g1_i1-w1_head:104-1279(+)
MARRPKMGPETYNIHINYSKDASVPNELPYLGWSYPYEYQHMVHQHWRSFSEPSLYYEIAFVIFFAVLLLTSLFGNGLVIWIFSTAKTLRTPSN
LLIINLAAFDLTMALNMPHYLINAVMRYFPGGDLACDIYAVLGGISGMGAAITNAFIAYDRYRTISNPLDGRLNYKQIIILIAISWLWTIPFSV
LPFLHIWGRYIPEGFLTSCSFDYLTDDDETRYFVRAMFIWAYCIPMIVICIYYTKLFLHVRQHEQMLADQAKKMNVKSLIAYPHNTGLSTELRI
AKAAMIIYLMYVFAWTPYATIALLGTFGYSHLITPFASMIPCCCAKIVSCLDPWIYAASHPKYRAVLASRLPFLGIQEKGNSITSSNDNGNING
GGSNDGDATTLSVIA*
>TRINITY_DN435_c0_g5_i1-w4_head|m.184 TRINITY_DN435_c0_g5_i1-w4_head|g.184 type:complete
len:376 TRINITY_DN435_c0_g5_i1-w4_head:228-1355(+)
MISQKIGIIQYLHGGPSLGSGHNISLAESVPAEIYHMVDPYWYQWPPMDQMWFGIIAFVITILGIMSLSGNFIVMYIFTSSKSLRTPSNMFVVN
LAFSDFMMMFTMFPPVVLNSFYGTWILGPLWCELYGLFGSLFGCVSIWSMTLIAYDRYCVIVKGLSAKPLTSSIAALRLLVVWIICLSWGLLPM
FGWNRYVPEGNMTACGTDYFAKDWWNRSYIIVYSLWVYFTPLLTIIFSYWHILKAVRAHEQAMREQAKKMNVSSLRNTETDKGKSVEIKLAKVA
LITITLWFMAWTPYTIINYAGIFESMHLSPLSTICGSIFAKANSIYNPIVYGLSHPKYKQVLREKMPCLVCGKDDNNADVRTQATGEVSESIA*
```

Journal of Experimental Biology • Supplementary information

References

Athanasiou, A., Aguila, M., Bellingham, J., Li, W., McCulley, C., Reeves, P.J., and Cheetham, M.E. (2018). The molecular and cellular basis of rhodopsin retinitis pigmentosa reveals potential strategies for therapy. *Progress in Retinal and Eye Research* **62**, 1-23.

Dibo, N., Yang, Y., Wu, X., and Meng, F. (2023). A brief review on deer keds of the genus *Lipoptena* (Diptera: Hippoboscidae). *Veterinary Parasitology* **313**, 109850.

Hallgren, J., Tsirigos, K.D., Pedersen, M.D., Almagro Armenteros, J.J., Marcatili, P., Nielsen, H., Krogh, A., and Winther, O. (2022). DeepTMHMM predicts alpha and beta transmembrane proteins using deep neural networks. *Biorxiv* 2022.04.08.487609.

Klopfenstein, D.V., Zhang, L., Pedersen, B.S., Ramírez, F., Warwick Vesztrocy, A., Naldi, A., Mungall, C.J., Yunes, J.M., Botvinnik, O., Weigel, M., et al. (2018). GOATOOLS: A Python library for Gene Ontology analyses. *Sci. Rep.* **8**, 10872.

Madeira F., Madhusoodanan, N., Lee, J., Eusebi, A., Niewielska, A., Tivey, A.R.N., Lopez, R., and Butcher, S. (2024). The EMBL-EBI Job Dispatcher sequence analysis tools framework in 2024. Nucleic Acids Research **52**(W1), W521–W525.

Rice, P., Longden, I., and Bleasby, A. (2000). EMBOSS: the European Molecular Biology Open Software Suite. *Trends Genet.* **16**(6):276-7.

Supek, F., Bošnjak, M., Škunca, N., and Šmuc, T. (2011). REVIGO summarizes and visualizes long lists of gene ontology terms. *PloS One* **6**(7), e21800.

Xiao, Y., Hsiao, T.H., Suresh, U., Chen, H.I.H., Wu, X., Wolf, S.E., and Chen, Y. (2014). A novel significance score for gene selection and ranking *Bioinformatics* **30**(6), 801-807.

Journal of Experimental Biology • Supplementary information
